# Nitric oxide-mediated modulation of reproductive resilience under cold stress in chickpea

**DOI:** 10.3389/fpls.2025.1679156

**Published:** 2025-11-07

**Authors:** Sarbjeet Kaur, Deeksha Padhiar, Uday Chand Jha, Sanjeev Kumar, Kamal Dev Sharma, Swarup Kumar Parida, Kadambot H. M. Siddique, P. V. Vara Prasad, Harsh Nayyar

**Affiliations:** 1 Department of Botany, Panjab University, Chandigarh, India; 2 Crop Improvement Division, Indian Institute of Pulses Research, Kanpur, India; 3 Department of Plant Sciences, Central University of Punjab, Bathinda, India; 4 Department of Agriculture Biotechnology, Chaudhary Sarwan Kumar (CSK) Himachal Pradesh Agricultural University, Palampur, India; 5 Department of Biotechnology, National Institute of Plant Genome Research, New, Delhi, India; 6 The University of Western Australia Institute (UWA) of Agriculture, The University of Western Australia, Perth, WA, Australia; 7 Sustainable Intensification Innovation Lab, Kansas State University, Manhattan, KS, United States

**Keywords:** nitric oxide, cold, stress, chickpea, SNP, pollen viability, antioxidant defense

## Abstract

Chickpeas are particularly sensitive to cold stress during the reproductive phase, which can significantly impair pod set and yield. This study examined the role of sodium nitroprusside (SNP), a nitric oxide (NO) donor, in mitigating cold-induced reproductive damage in cold-tolerant (CT) and cold-sensitive (CS) chickpea genotypes. After 100 days of outdoor growth, plants were subjected to cold stress (15/8°C day/night; 12 h photoperiod) for 21 days in walk-in growth chambers during the reproductive stage of development. Control plants were maintained at 25/15°C day/night temperature. SNP treatment (1 mM) was applied exogenously each time, first two days prior to stress onset and then at seven-day intervals (three applications total). Cold stress significantly lowered endogenous NO levels in leaves, anthers, and ovules, particularly in CS genotypes, thereby leading to reduced pollen viability and germination. SNP treatment restored NO and improved reproductive performance, with stronger responses in the CS than the CT genotype. For instance, pollen germination increased by 57.9% in CS versus 17.6% in CT, and pollen viability increased by 28.0% and 13.1%, respectively. Enhanced anther function resulted in a 157.2% increase in pod set and 62.0% higher seed yield in CS. SNP also improved physiological traits, including a 43.9% increase in cellular viability, 18.6% in stomatal conductance, and 41.9% in chlorophyll content in CS genotypes. Cryoprotectants (proline, trehalose, and sucrose) accumulated in anthers, reinforcing cold resilience, while oxidative stress was simultaneously alleviated through reduced malondialdehyde, hydrogen peroxide, and electrolyte leakage, together with the upregulation of both enzymatic (superoxide dismutase (SOD), catalase (CAT), ascorbate peroxidase (APx), and glutathione reductase (GR)) and non-enzymatic (ascorbic acid (ASC) and reduced glutathione (GSH)) components. Notably, CS genotypes showed more pronounced improvements from SNP application than CT genotypes, particularly in terms of reproductive success and yield-related traits. These findings highlight the potential of NO donors, such as SNP, to enhance cold tolerance in chickpeas, with promising implications for safeguarding productivity under low-temperature stress, especially in sensitive cultivars.

## 1 Introduction

Chickpeas (*Cicer arietinum* L.) are a major legume crop cultivated worldwide, particularly in semi-arid regions, where they serve as a key source of dietary protein and support sustainable agricultural systems (Gaur et al., 2015). According to the Food and Agriculture Organization of the United Nations (FAO), 2025, the average global production of chickpeas is 16.51 million tons. India accounts for the largest chickpea area (10.47 Mha) and production (12.27 Mt), whereas Australia recorded the highest yield (3301.7 kg/ha) (FAOSTAT 2025). Other major producers include Pakistan (0.84 Mha, 0.24 Mt), Myanmar (0.32 Mha, 0.41 Mt), Türkiye (0.46 Mha, 0.58 Mt), Ethiopia (0.21 Mha, 0.45 Mt), Canada (0.13 Mha, 0.14 Mt), Mexico (0.075 Mha, 0.14 Mt), the United States of America (USA) (0.15 Mha, 0.21 Mt), and Russia (0.40 Mha, 0.53 However, chickpeas are highly sensitive to cold stress, typically defined as temperatures below 20/10°C (day/night), especially during their reproductive stage. This critical phase often coincides with low temperatures in many production regions (Clarke and Siddique, 2004; Nayyar et al., 2005; Berger et al., 2012). Exposure to cold during reproduction can lead to substantial yield losses owing to flower abortion, reduced pod set, and impaired seed development (Croser et al., 2003; Nayyar et al., 2005; Rani et al., 2020). These yield penalties are primarily attributed to impaired pollen viability, ineffective fertilization, and oxidative damage to floral tissues, which interfere with normal reproductive processes (Kiran et al., 2019; Rani et al., 2021; Kaur et al., 2022).

With climate change increasing the frequency and severity of extreme weather events, improving cold stress tolerance in chickpeas has become an urgent breeding and agronomic priority. One promising strategy involves the application of nitric oxide (NO), a versatile signaling molecule that plays a key role in modulating plant responses to abiotic stressors. Nitric oxide (NO) has been extensively studied for its ability to enhance stress tolerance in various species, including cold stress (Siddiqui et al., 2011; Puyaubert and Baudouin, 2014; Fancy et al., 2017). It acts as a regulator of physiological and molecular responses by modulating antioxidant defenses, maintaining cellular redox balance, and activating the expression of stress-responsive genes (Esim et al., 2014; Zhang et al., 2019; Wang et al., 2021). In cold-stressed tea plants, NO elevates the levels of protective metabolites, including gamma-aminobutyric acid (GABA), proline, and sugars (Wang et al., 2021). In wheat, NO application preserves the integrity of the photosynthetic apparatus and stabilizes the cell membranes under cold stress (Feng et al., 2021). The protective role of NO is multifaceted; it can directly scavenge reactive oxygen species (ROS), enhance the activity of antioxidant enzymes, and mitigate oxidative damage to cellular components, including lipids, proteins, and nucleic acids (Fan et al., 2015). Additionally, NO interacts with other signaling molecules to activate cold-responsive pathways and gene expression, thereby safeguarding cellular structures under stress (Feng et al., 2021; Wu et al., 2022).

NO also plays a vital role in plant reproduction, particularly in regulating pollen tube growth and guidance, which are essential for successful fertilization. This function is thought to be mediated through the cyclic guanosine monophosphate (cGMP) signaling pathway (Prado et al., 2004). Although the role of NO in reproductive development under normal conditions is well established, its involvement in protecting reproductive structures under cold stress remains largely unexplored. Most existing studies have focused on NO-mediated protection in vegetative tissues, leaving a significant gap in our understanding of its specific role in safeguarding fertility and yield during low-temperature stress in reproductive tissues.

To address this gap, the present study investigated the potential of exogenously applied NO to preserve reproductive function in chickpeas under cold stress. We hypothesized that endogenous NO in both leaves and reproductive tissues plays a critical role in maintaining fertility during cold stress. Using cold-tolerant and cold-sensitive chickpea genotypes, we evaluated the efficacy of sodium nitroprusside (SNP), a widely used NO donor, in mitigating cold-induced reproductive damage in plants. Specifically, we assessed the effects of SNP treatment on flower retention, pollen viability, pod set and seed yield. We also examined its impact on oxidative damage in anthers and ovules, which are key determinants of reproductive success. Our findings demonstrate that NO enhances cold resilience by reducing oxidative stress, increasing cryoprotective compounds, and improving the functionality of reproductive tissues. These results suggest that NO application could be a promising strategy for improving cold tolerance in chickpea and other sensitive legume crops, ultimately contributing to more stable yields under adverse environmental conditions. Therefore, this study aimed to (i) determine how endogenous NO levels and related enzymes (NOS and NR) respond in chickpea genotypes under cold stress, (ii) evaluate the efficacy of exogenous SNP application in modulating physiological, biochemical, and reproductive traits, and (iii) assess whether SNP can improve pod set and yield, particularly in cold-sensitive genotypes.

## 2 Materials and methods

### 2.1 Preliminary experiment: plant raising and stress imposition

#### 2.1.1 Plant material and growth conditions

A preliminary experiment was conducted to evaluate endogenous NO levels and the activities of nitric oxide synthase (NOS) and nitrate reductase (NR) in the leaves, anthers, and ovules of cold-tolerant (CT, ICC 17258) and cold-sensitive (CS, GPF 2) chickpea genotypes under cold stress. Seeds were surface sterilized with 0.1% mercuric chloride for 2 min, rinsed twice with distilled water, inoculated with *Mesorhizobium cicero*, and sown in pots containing 8 kg of sandy loam soil (63.4% sand, 24.6% silt, 12% clay; pH 7.1) mixed with sand at a ratio of 3:1. The soil was amended with farmyard manure and tricalcium phosphate (10 mg kg^−1^) (Awasthi et al., 2014). The soil organic carbon content was 1.4–6.1 g/kg, with nutrient availability of 54, 43, and 158 kg ha^−1^ for N, P, and K, respectively.

#### 2.1.2 Growth regime and cold stress treatment

Seeds were sown in the first week of November 2022. Plants were raised outdoors for 50 days under ambient conditions (26.9/16.6°C day/night; 1,300–1,500 μmol m^−2^ s^−1^ light; 60–70% RH). At reproductive onset (bud to pod formation), plants were exposed to 25/15°C, 12 h photoperiod, in a walk-in chamber. Another set of plants was exposed to cold stress (15/8°C (12 h photoperiod; 500 μmol m^−2^ s^−1^ light; 65–70% relative humidity (RH)), for 21 days. For this treatment, the temperature of the chamber was reduced by 2°C per day until the desired low temperature was reached. The control plants were maintained at 25/15°C under identical conditions. Recovery was achieved by raising the temperature stepwise (2°C per day) to 32/22°C for both control and stressed plants until they reached maturity.

#### 2.1.3 Sampling and measurements

After 21 days of cold stress, NO, NOS, and NR activity were assayed in all three organs. Three biological replicates were analyzed for each genotype × treatment combination.

##### 2.1.3.1 Endogenous nitric oxide levels

Fresh leaf tissue (approximately 0.5 g) was homogenized in 3 mL of ice-cold 50 mM acetic acid buffer (pH 3.6) containing 4% zinc diacetate using a mortar and a pestle. The homogenate was centrifuged at 10,000 *× g* for 15 min at 4°C, and the resulting supernatant was collected. The pellet was re-extracted with 1 mL of the same buffer and centrifuged again under the same conditions. The supernatants were combined, and 0.1 g of charcoal was added to the mixture. The solution was vortexed and filtered to obtain the final extract. Nitrite, a stable oxidation product of NO, was quantified by mixing 1 mL of the filtrate with 1 mL of Griess reagent and incubating the mixture at room temperature for 30 min. The absorbance was measured at 540 nm using a spectrophotometer. The nitric oxide concentration was calculated using a standard curve generated with sodium nitrite (NaNO_2_), following the protocol described by Zhou et al. (2005).

##### 2.1.3.2 Nitrate reductase and nitric oxide synthase activities

Fresh plant tissue was homogenized in 5 mL of 10 mM phosphate buffer (pH 7.0) containing 4% (w/v) polyvinylpyrrolidone (PVP) and 1 mM ethylenediaminetetraacetic acid (EDTA). The homogenate was centrifuged at 12,000 *× g* for 15 min at 4°C, and the resulting supernatant was used as the crude enzyme extract.

For protein extraction, samples were homogenized in a buffer containing 100 mM HEPES–KOH (4-(2-hydroxyethyl)-1-piperazineethanesulfonic acid buffered with potassium hydroxide) (pH 7.5), 1 mM EDTA, 10% (v/v) glycerol, 5 mM 1,4-dithiothreitol (DTT), 0.5 mM phenylmethyl sulfonyl fluoride (PMSF), 0.1% (v/v) Triton X-100, 1% PVP, and 20 µM flavin adenine dinucleotide (FAD). After centrifugation at 12,000 *× g* for 20 min at 4°C, the supernatant was collected for spectrophotometric assays of NR and NOS activity at 520 nm and 340 nm, respectively, following the modified protocols of Sun et al. (2014) and Zhao et al. (2009).

To assess NR activity, 250 µL of enzyme extract was mixed with 250 µL of pre-warmed (25°C) assay buffer containing 50 mM HEPES–KOH (pH 7.5), 10 mM MgCl_2_ (magnesium chloride), 1 mM DTT, 2 mM KNO_3_ (potassium nitrate), and 200 µM NADH (nicotinamide adenine dinucleotide). The reaction was carried out at 30°C for 30 min and terminated by adding 50 µL of 0.5 M zinc acetate. Nitrite formation was measured by adding 1 mL of 1% sulfanilamide in 3 M HCl (hydrochloric acid) and 1 mL of 0.02% N-(1-naphthyl) ethylenediamine in 0.2 M HCl. For the nitrite assay, 1.5 mL of the filtrate was mixed with an equal volume of Griess reagent containing 1% sulfanilamide and 0.1% N-1-naphthyl ethylenediamine dihydrochloride dissolved in 5% phosphoric acid (H_3_PO_4_). The mixture was incubated at room temperature for 30 min to allow the colorimetric reaction to occur. Absorbance was measured at 540 nm, and the nitrite concentration was calculated using a standard curve prepared with sodium nitrite (NaNO_2_).

NOS activity was measured in a 1 mL reaction mixture containing 100 mM phosphate buffer (pH 7.0), 1 mM L-arginine, 2 mM MgCl_2_, 0.3 mM CaCl_2_, 4 µM tetrahydrobiopterin (BH_4_), 1 µM FAD, 1 µM flavin mononucleotide (FMN), 0.2 mM DTT, 0.2 mM NADPH, and 200 µL of protein extract. The rate of NADPH oxidation was monitored by recording the decrease in absorbance at 340 nm for 5 min. NOS activity was calculated using the extinction coefficient of NADPH (ϵ = 6.22 mM^−1^ cm^−1^).

### 2.2 Optimization of exogenous SNP concentration under low temperatures

A follow-up preliminary experiment was conducted to identify the optimal SNP concentration for alleviating cold stress. Plants of a CT (ICC 17258) and CS (GPF 2) chickpea genotype were grown outdoors for 50 d under ambient conditions, with temperatures ranging from 16.6 to 26.9°C, a light intensity of 1,300–1,500 μmol m^−2^ s^−1^, and 60–70% relative humidity. Seed treatment with *Rhizobium* prior to sowing and pot soil details are provided in Section 2.1. At the start of the reproductive phase, some plants were maintained in a chamber at 25/15°C (12 h photoperiod) to serve as a control group. Another group was subjected to a 21-day cold stress treatment, during which the chamber temperature was gradually decreased by 2°C per day until it reached the desired low temperatures (18/8°C, 15/8°C, and 13/7°C day/night) with a light intensity of 500 μmol m ^−2^ s ^−1^. SNP (0.5, 1, and 1.5 mM) was applied as a foliar spray using 20 mL per plant (with 0.05% Tween-20 as a surfactant) to 50-day-old plants. The first application was administered 2 days prior to cold stress, followed by two additional sprays at 7-day intervals. Following this, the temperature for both groups was slowly raised by 2°C per day until it reached 32/22°C, and the plants were maintained at this temperature until they matured. The pod set, defined as the percentage of flowers that developed into pods, was recorded at maturity. Each treatment was applied in triplicate for both genotypes under control and cold stress conditions, and samples for analysis were collected from these biological replicates.

### 2.3 In-depth experiments

Based on preliminary optimization (Section 2.2), **1 mM SNP** was identified as the most effective concentration, particularly under moderate cold stress (15/8°C), and was therefore used in subsequent detailed experiments.

#### 2.3.1 Experimental design and plant material

Four chickpea genotypes were selected based on their contrasting responses to cold stress in prior screenings: two cold-tolerant (CT: ICC 17258 and ICC 16349) and two cold-sensitive (CS: ICC 15567 and GPF 2). Seeds were obtained from ICRISAT (Hyderabad, India) and PAU (Ludhiana, India). Plants were raised as described in Section 2.1.

#### 2.3.2 Treatments and SNP application

Plants were exposed to two temperature regimes:

- Control: 25/15°C (day/night, 12 h photoperiod), and
- Low-temperature stress (LT): 15/8°C (day/night, 12 h photoperiod).

Within each regime, the plants were divided into two groups: untreated and treated with SNP (1 mM). SNP was applied as a foliar spray with Tween 20 surfactant, at a volume of ~20 mL per plant, two days prior to cold stress induction, followed by two further applications at 7-day intervals.

The resulting treatments were as follows: Control, Control + SNP (1 mM), LT, and LT + SNP (1 mM).

#### 2.3.3 Sampling and replication

Each treatment was performed in triplicate using independent biological sets. Leaves, anthers, and ovules were sampled after 21 d of cold stress for physiological and biochemical assays, whereas yield-related traits (flowering, pod set, and seed yield) were assessed at physiological maturity.

#### 2.3.4 Endogenous nitric oxide

Endogenous NO levels were measured as described in Section 2.1.1.

#### 2.3.5 Physiological and biochemical measurements

##### 2.3.5.1 Electrolyte leakage

Membrane damage was estimated by measuring electrolyte leakage (EL) from the leaf discs. The samples were incubated in deionized water at 25°C for 12 h, and the initial conductivity (C1) was recorded. After heating at 80°C for 10 min, the final conductivity (C2) was measured. EL (%) was calculated as (C1/C2) × 100 (Kaushal et al., 2013).

##### 2.3.5.2 Cellular viability

Cell viability was assessed using the TTC reduction assay based on mitochondrial activity. Fresh tissues were incubated with 0.5% TTC in phosphate buffer (pH 7.4) at 25°C in the dark. Formazan was extracted in ethanol and absorbance read at 530 nm. The results were expressed as absorbance g^−1^ FW (Steponkus and Lanphear, 1967).

##### 2.3.5.3 Relative leaf water content

Relative leaf water content (RLWC) was used to assess the leaf water status (Barrs and Weatherley, 1962). Fresh leaf samples (500 mg) were immersed in distilled water for 2 h, blotted dry with filter paper, and weighed to determine the turgid weight (TW). The samples were then oven-dried at 110°C for 24 h to obtain the dry weight (DW). RLWC was calculated as:

RLWC (%)=(FW−DW)/(TW−DW)×100, where FW is the fresh weight.

##### 2.3.5.4 Stomatal conductance

Stomatal conductance (SC) was measured on fully expanded leaves using a portable leaf porometer (Decagon Devices, Pullman, Washington, USA). Values are expressed in millimoles per square meter per second (mmol m^−2^ s^−1^).

##### 2.3.5.5 Chlorophyll content

Chlorophyll was extracted from 500 mg of fresh leaf tissue using 80% acetone. The extract was centrifuged at 5,702 *× g* for 15 min, and the supernatant was collected for analysis. Absorbance was measured at 666 and 653 nm using a spectrophotometer. The total chlorophyll content was calculated using the formula described by Lichtenthaler and Wellburn (1983).

##### 2.3.5.6 Chlorophyll fluorescence

The maximum quantum efficiency of photosystem II (Fv/Fm) was measured to assess the photochemical efficiency of the leaves using a chlorophyll fluorometer (OS1-FL; Opti-Sciences, Hudson, New Hampshire, USA).

#### 2.3.6 Oxidative stress and antioxidants

##### 2.3.6.1 Malondialdehyde concentration

MDA, a marker of lipid peroxidation, was quantified using TBA–MDA adducts. Tissue extracts in TCA were reacted with TBA in TCA, heated at 95°C, cooled, centrifuged, and the absorbance was recorded at 532 nm. MDA was calculated using an extinction coefficient of 155 mM^−1^ cm^−1^ (Heath and Packer, 1968).

##### 2.3.6.2 Hydrogen peroxide

H_2_O_2_ was extracted in 80% acetone, reacted with titanium reagent and ammonia, centrifuged, and the pellet was dissolved in H_2_SO_4_. Absorbance was measured at 410 nm, and H_2_O_2_ content was calculated using an extinction coefficient of 0.28 mmol^−1^ cm^−1^ (Mukherjee and Choudhuri, 1983).

#### 2.3.7 Antioxidant enzyme activities

- Superoxide dismutase (SOD): Activity was measured by monitoring the inhibition of NBT photoreduction in a riboflavin–methionine system at 560 nm. Results were expressed as units mg^−1^ protein (Dhindsa and Matowe, 1981).
- Catalase (CAT): Activity was measured by monitoring the decomposition of H_2_O_2_ at 410 nm (ϵ = 40 mM^−1^ cm^−1^) and expressed as mmol min^−1^ mg^−1^ of protein (Teranishi et al., 1974).
- Ascorbate peroxidase (APX): Activity was measured by monitoring ascorbate oxidation at 290 nm (ϵ = 2.8 mM^−1^ cm^−1^) and expressed as mmol min^−1^ mg^−1^ protein (Nakano and Asada, 1981).
- Glutathione reductase (GR): Activity was measured by following NADPH oxidation at 340 nm and expressed as mmol min^−1^ mg^−1^ protein (Mavis and Stellwagen, 1968).

#### 2.3.8 Non-enzymatic antioxidants

- Ascorbic acid (ASC): Extracted in TCA, reacted with DNPH and thiourea, developed with H_2_SO_4_, and absorbance was measured at 530 nm. ASC content was quantified from a standard curve and expressed as mg g^−1^ DW (Mukherjee and Choudhuri, 1983).
- Reduced glutathione (GSH): Extracted in metaphosphoric acid, reacted with DTNB, NADPH, and GR enzyme, and absorbance was measured at 412 nm. The GSH content was determined from a standard curve and expressed as nmol g^−1^ DW (Griffith, 1980).

#### 2.3.9 Osmolyte and sugar analysis

##### 2.3.9.1 Proline

The proline content was estimated using the ninhydrin method. Fresh tissue was homogenized in 3% sulfosalicylic acid, centrifuged, and reacted with acid ninhydrin. After heating and toluene extraction, the absorbance was measured at 520 nm. The results were expressed as µmol g^−1^ DW (Bates et al., 1973).

##### 2.3.9.2 Trehalose

Trehalose was quantified using the anthrone-sulfuric acid method. The samples were hydrolyzed in HCl, neutralized, and reacted with anthrone. Absorbance was measured at 620 nm, and the trehalose concentration was determined using a standard curve (Trevelyan et al., 1952).

##### 2.3.9.3 Sucrose

The sucrose content was determined using a resorcinol reagent. Tissue extracts were hydrolyzed, reacted with resorcinol-HCl, and the absorbance was measured at 480 nm. The results are expressed as mg g^−1^ DW (van Handel, 1968).

#### 2.3.10 Reproductive trait assessment

Pollen viability, germination, and ovule viability were evaluated according to established protocols.

- Pollen viability: Assessed using Alexander’s stain (Alexander, 1969). Viable pollen was stained purple, and non-viable pollen remained green.
- Pollen germination: Evaluated *in vitro* using a germination medium containing sucrose and boric acid. Germinated pollen was counted microscopically, as described by Brewbaker and Kwack (1963).
- Ovule viability was assessed by fluorescein diacetate (FDA) staining, with viable ovules fluorescing under UV light (Heslop-Harrison and Heslop-Harrison, 1970).
- Stigma receptivity: Determined using the hydrogen peroxide bubbling test, in which active peroxidases in receptive stigmas produce effervescence (Shivanna and Rangaswamy, 1992).
- Pod set: Calculated as the percentage of flowers that developed into mature pods at physiological maturity (Sharma and Nayyar, 2014).

### 2.4 Statistical analysis

A preliminary study assessed endogenous NO, NOS, and NR activities in two contrasting chickpea genotypes across three organs under control and cold stress conditions **(Section 2.1).** Each genotype was represented by three pots (each containing two plants) with three biological replicates per treatment (nine plants in total). Data were analyzed using a three-way ANOVA (Genotype × Treatment × Organ) in RStudio (R Core Team, 2023) to evaluate main and interactive effects. As the three-way interaction effects (Genotype × Treatment × Organ) showed inconsistent significance across traits, *post hoc* Tukey’s HSD test (p< 0.05) was applied within each organ to compare genotype × treatment combinations using the *emmeans* package (Lenth, 2023), and compact letter displays (CLD) were generated with *multcomp* (Hothorn et al., 2008) (**Supplementary Figure 1**). Summary statistics were processed using *dplyr* (Wickham et al., 2023), and visualizations were created with *ggplot2* (Wickham, 2016). ANOVA tables, including degrees of freedom, mean squares, and significance levels, are provided in **Supplementary Table 1**.

The subsequent experiment **(Section 2.2**) evaluated the effect of different SNP concentrations at various temperatures on pod set (%). Expanded to include two genotypes, four temperature regimes, and four SNP concentrations (32 treatment combinations). Data were analyzed using three-way ANOVA (Genotype × Temperature × SNP). *Post hoc* comparisons were performed using Tukey’s test (emmeans), and CLD was generated with *the multcomp* package. Data handling with *dplyr*, plots with *ggplot2*. ANOVA tables including df, sums of squares, mean squares, and significance levels are provided in **Supplementary Table 2**.

**Figure 1:**
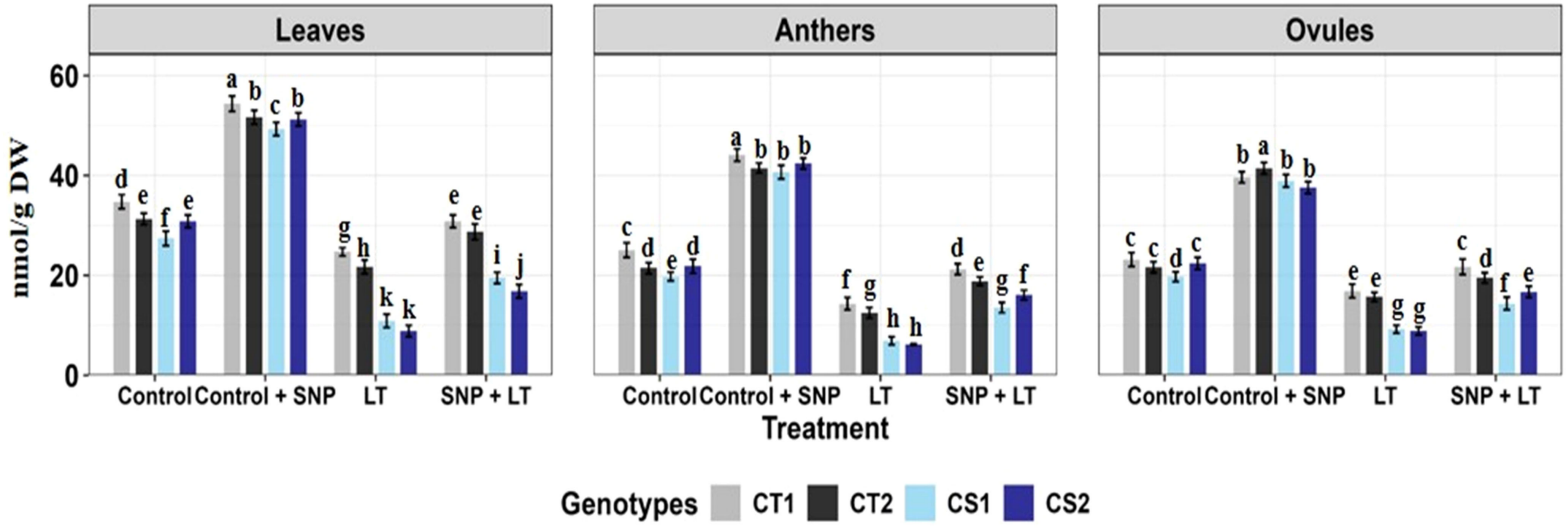
Endogenous NO levels (nmol/g DW) in three organs (leaves, anthers, ovules) of four contrasting genotypes (CT1: ICC 17258; CT2: ICC 16349; CS1: ICC 15567; CS2: GPF-2) under different treatments: Control, Control + SNP, Low Temperature (LT), and SNP + LT. Data represent mean ± SE (n = 3). Different lowercase letters indicate significant differences among genotype *treatment interaction according to Tukey's test (p<0.05), within each organ.

**Figure 2:**
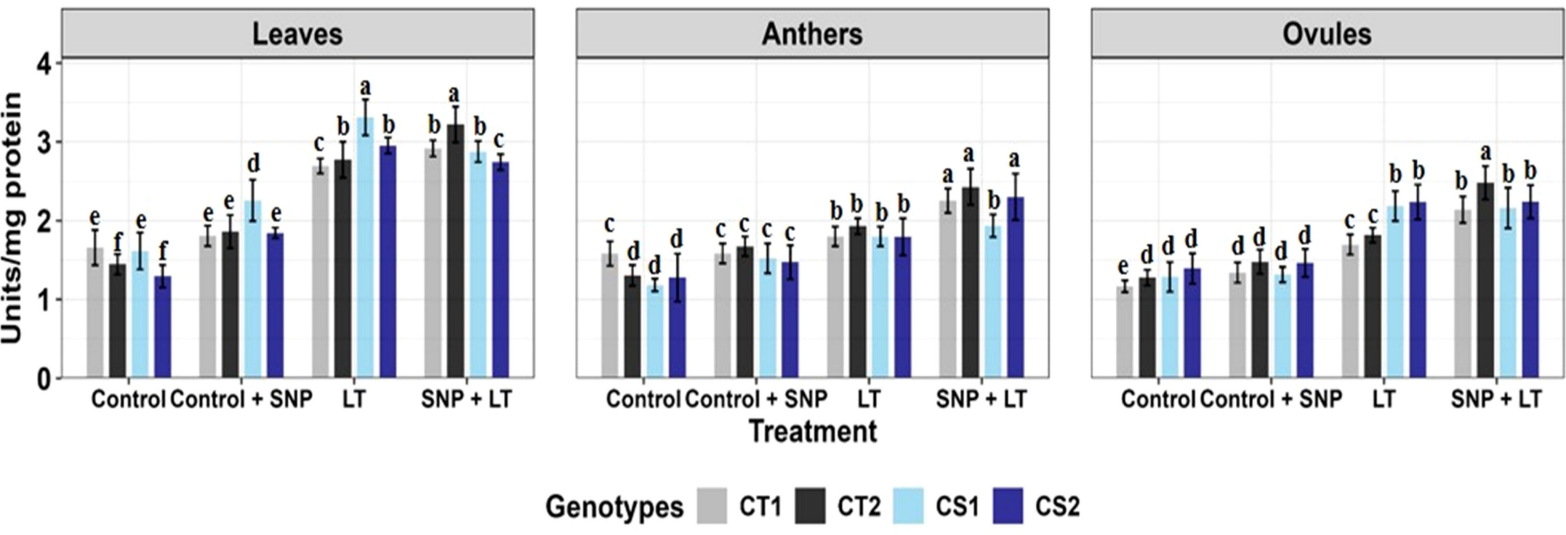
Superoxide dismutase activity in three organs (leaves, anthers, ovules) of four contrasting genotypes (CT1: ICC 17258, CT2: ICC 16349, CS1: ICC 15567, CS2: GPF-2) under different treatments: Control, Control + SNP, Low Temperature (LT), and SNP + LT. Data represent mean + SE (n = 3). Different lowercase letters indicate significant differences among genotype *treatment interaction according to Tukey's test (p<0.05), within each organ.

**Figure 3:**
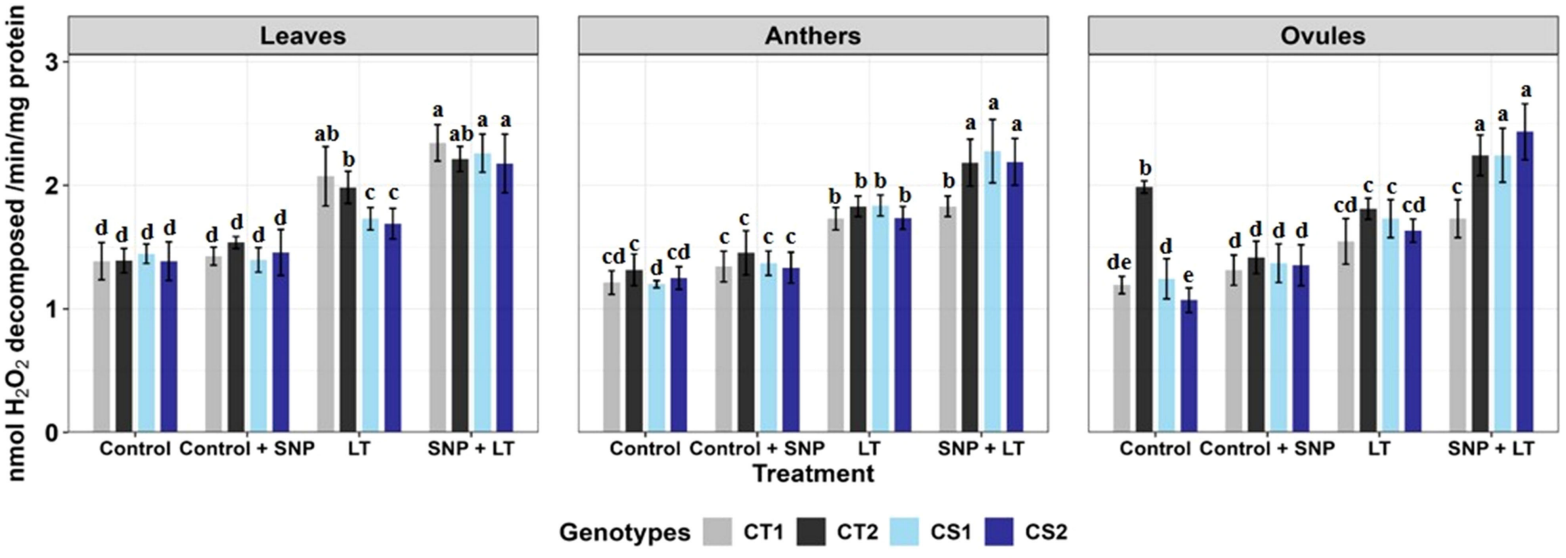
Catalase activity in three organs (leaves, anthers, ovules) of four contrasting genotypes (CT1: ICC 17258, CT2: ICC 16349, CS1: ICC 15567, CS2: GPF-2) under different treatments: Control, Control + SNP, Low Temperature (LT), and SNP + LT. Data represent mean ± SE (n =3). Different lowercase letters indicate significant differences among genotype *treatment interaction according to Tukey's test (p<0.05), within each organ.

**Figure 4:**
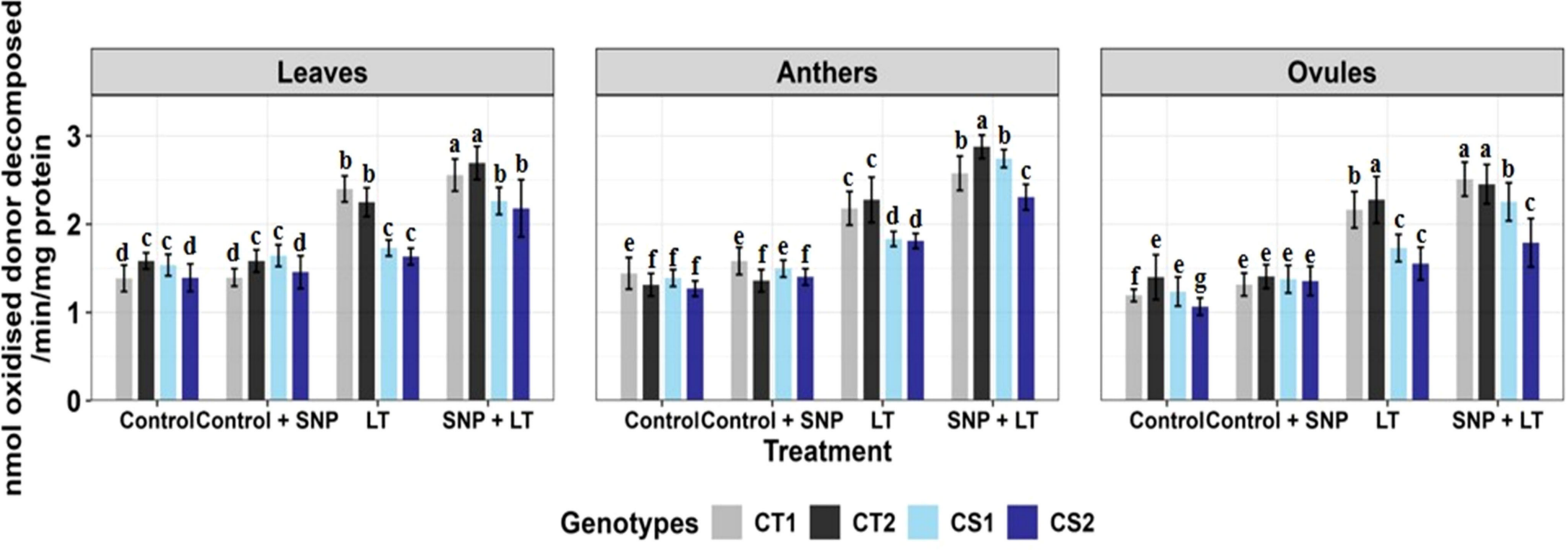
Ascorbate activity in three organs (leaves, anthers, ovules) of four contrasting genotypes (CT1: ICC 17258, CT2: ICC 16349, CS1: ICC 15567, CS2: GPF-2) under different treatments: Control, Control + SNP, Low Temperature (LT), and SNP + LT. Data represent mean ± SE (n =3). Different lowercase letters indicate significant differences among genotype *treatment interaction according to Tukey's test (p<0.05), within each organ.

**Figure 5:**
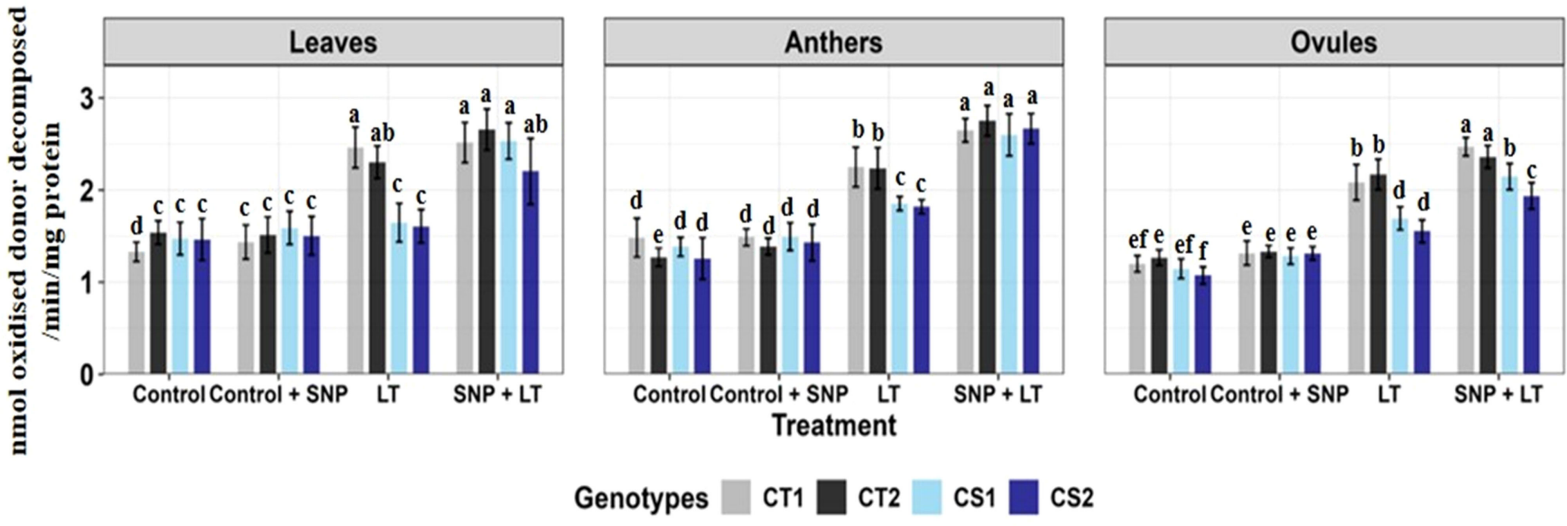
Glutathione reductase in three organs (leaves, anthers, ovules) of four contrasting genotypes (CT1: ICC 17258, CT2: ICC 16349, CS1: ICC 15567, CS2: GPF-2) under different treatments: Control, Control + SNP, Low Temperature (LT), and SNP + LT. Data represent mean ± SE (n = 3). Different lowercase letters indicate significant differences among genotype *treatment interaction according to Tukey's test (p<0.05), within each organ.

**Figure 6:**
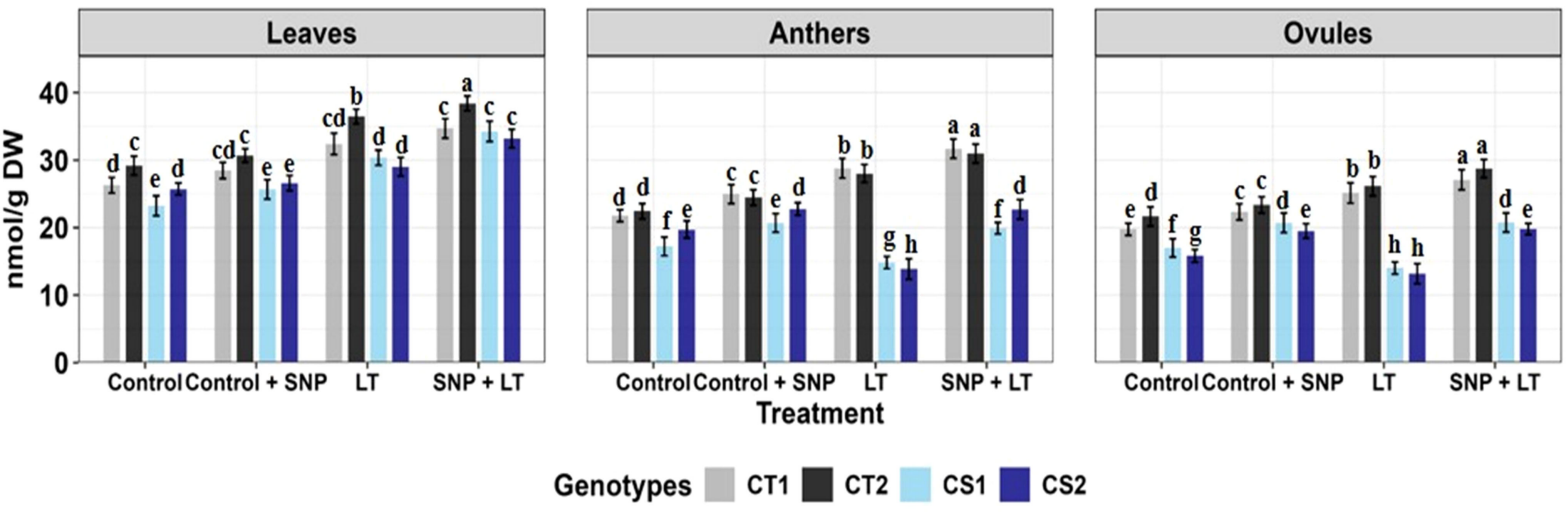
Ascorbic acid in three organs (leaves, anthers, ovules) of four contrasting genotypes (CT1: ICC 17258, CT2: ICC 16349, CS1: ICC 15567, CS2: GPF-2) under different treatments: Control, Control + SNP, Low Temperature (LT), and SNP + LT. Data represent mean ± SE (n = 3). Different lowercase letters indicate significant differences among genotype *treatment interaction according to Tukey's test (p <0.05), within each organ.

**Figure 7:**
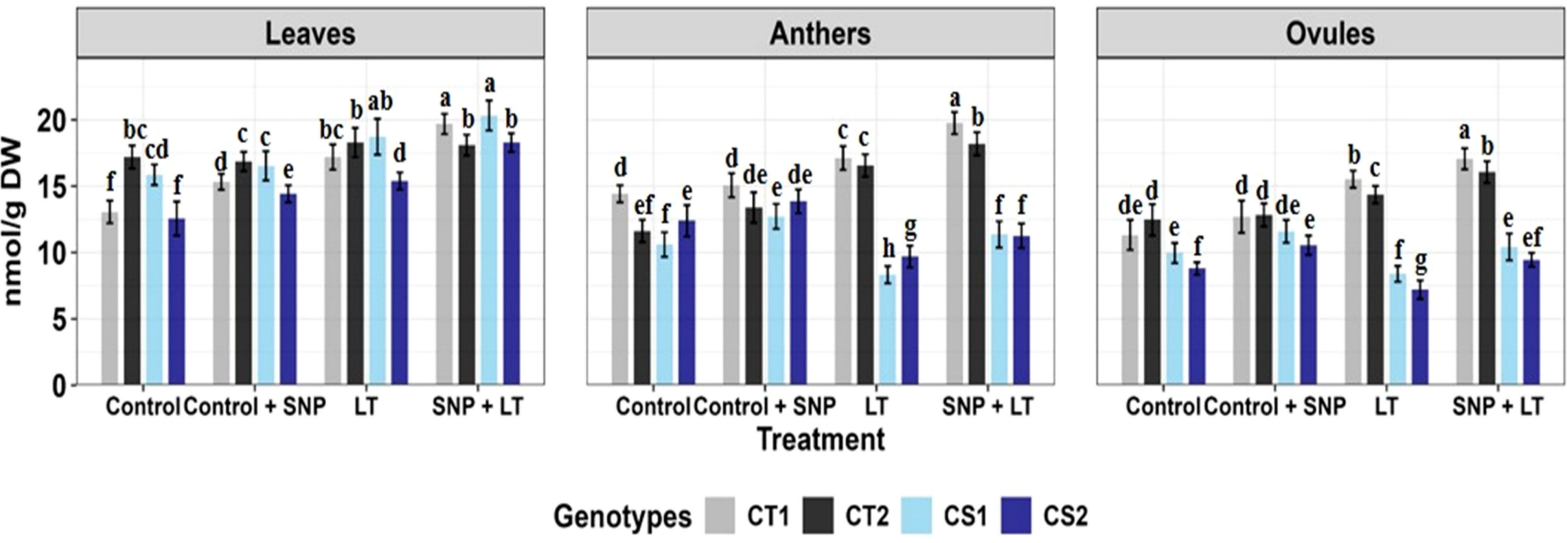
Reduced glutathione in three organs (leaves, anthers, ovules) of four contrasting genotypes (CT1: ICC 17258, CT2: ICC 16349, CS1: ICC 15567, CS2: GPF-2) under different treatments: Control, Control + SNP, Low Temperature (LT), and SNP + LT. Data represent mean ± SE (n = 3). Different lowercase letters indicate significant differences among genotype *treatment interaction according to Tukey's test (p<0.05), within each organ.

**Figure 8:**
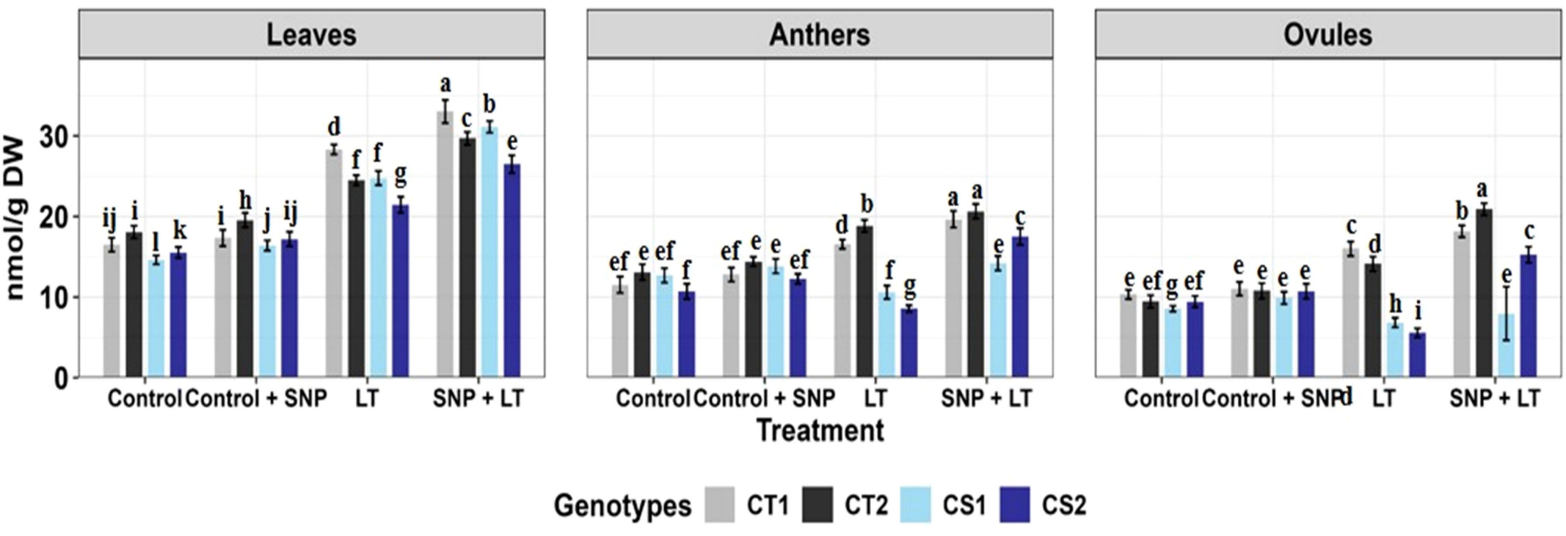
Proline content in three organs (leaves, anthers, ovules) of four contrasting genotypes (CT1: ICC 17258, CT2: ICC 16349, CS1: ICC 15567, CS2: GPF-2) under different treatments: Control, Control + SNP, Low Temperature (LT), and SNP + LT. Data represent mean + SE (n=3). Different lowercase letters indicate significant differences among genotype *treatment interaction according to Tukey's test (p<0.05), within each organ.

**Figure 9:**
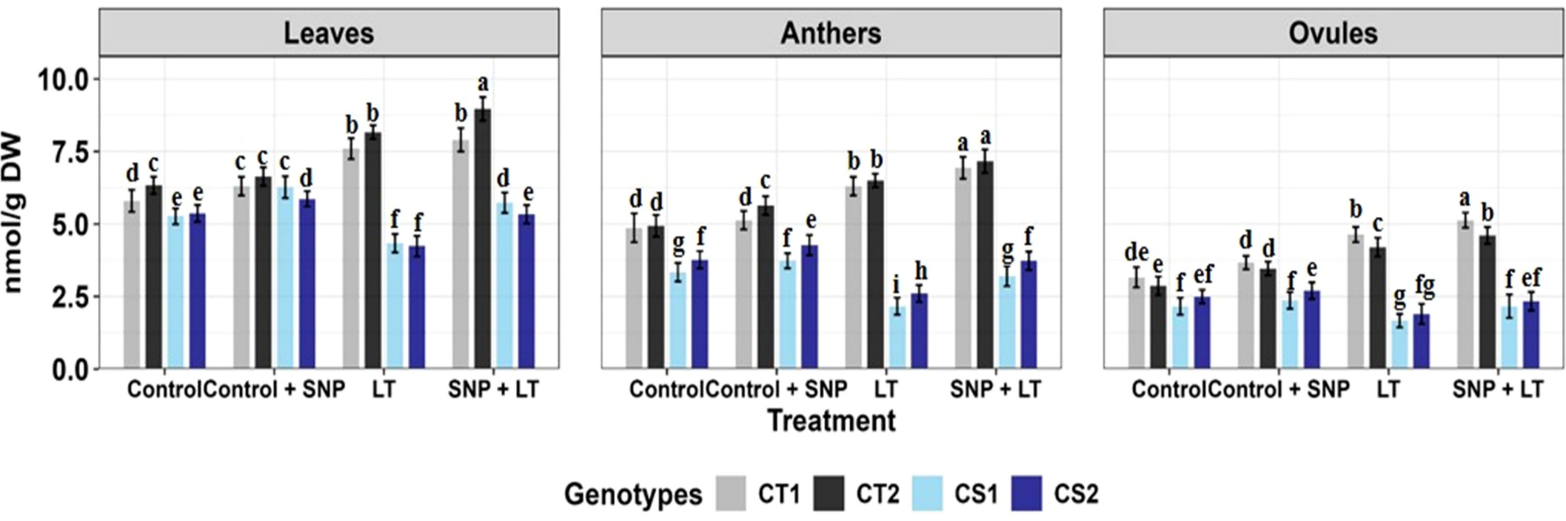
Trehalose content in three organs (leaves, anthers, ovules) of four contrasting genotypes (CT1: ICC 17258, CT2: ICC 16349, CS1: ICC 15567, CS2: GPF-2) under different treatments: Control, Control + SNP, Low Temperature (LT), and SNP + LT. Data represent mean ± SE (n = 3). Different lowercase letters indicate significant differences among genotype *treatment interaction according to Tukey's test (p<0.05), within each organ.

**Figure 10:**
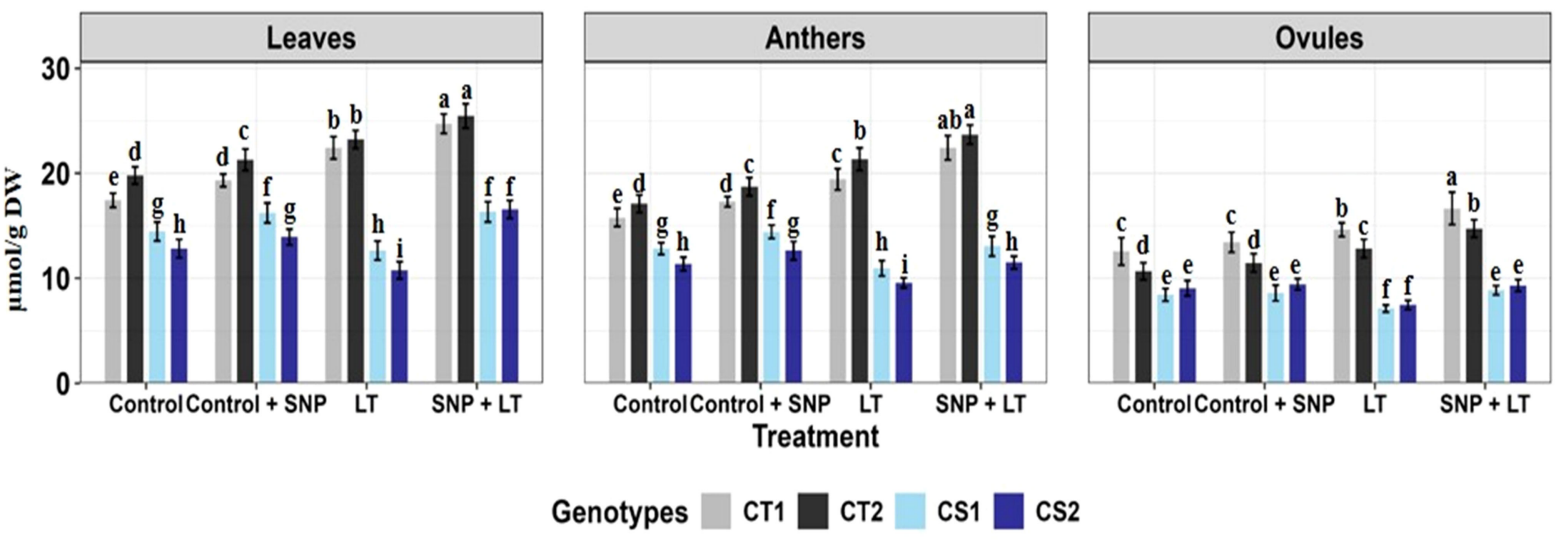
Sucrose content in three organs (leaves, anthers, ovules) of four contrasting genotypes (CT1: ICC 17258, CT2: ICC 16349, CS1: ICC 15567, CS2: GPF-2) under different treatments: Control, Control + SNP, Low Temperature (LT), and SNP + LT. Data represent mean ± SE (n = 3). Different lowercase letters indicate significant differences among genotype *treatment interaction according to Tukey's test (p<0.05), within each organ.

Based on these results, the selected treatment (1 mM SNP) was further evaluated using a completely randomized block design (CRBD) comprising four contrasting genotypes, four treatments, and three organs (leaves, anthers, and ovules) **(Section 2.3).** Each genotype was tested in three independent replicates. For physiological and biochemical traits, one plant per replicate was analyzed (n = 3), while for yield traits, three plants per replicate were used (n = 9 per genotype per treatment). Data for physiological and biochemical traits were analyzed using a three-way ANOVA (Genotype × Treatment × Organ). As the three-way interaction effects (Genotype × Treatment × Organ) showed inconsistent significance across traits, Tukey’s HSD test (p< 0.05) was applied within each organ to compare genotype × treatment combinations, ensuring consistency across all graphical (**Figures 1**, **2**, **3**, **4**, **5**, **6**, **7**, **8**, **9**, **10**) and tabulated representations (**Tables 1**, **2**, **3**, **4**). For traits not measured across all organs (leaf injury, reproductive, and yield), two-way ANOVA (Genotype × Treatment) was applied, followed by the Tukey test. Data handling and summary statistics were carried out using *dplyr*, and visualizations were generated with *ggplot2*. Complete ANOVA outputs are provided in **Supplementary Table 3**.

**Table 1:** Effect of cold stress on physiological parameters in various organs and contrasting chickpea genotypes under four treatments (control, control+SNP, LT, SNP+LT) and with percentage change.

*Traits*	*Tissue*	*Genotypes*	*Control*	*Control+SNP*	*%Change*	*lt*	*Snp+lt*	*% change*
*EL*	**Leaves**	**CT1**	11.7±1.07g	10.6±0.86h	- 9.4	21.4±1.15d	15.6±0.87f	-27.1
		**CT2**	13.1±0.89fg	12.3±0.90g	- 6.1	23.4±1.21c	13.5±0.89fg	-42.3
		**CS1**	12.8±0.86g	11.6±0.81g	- 9.4	27.8±1.16a	18.7±1.09e	-32.7
		**CS2**	14.3±0.95f	12.4±1.03g	- 13.3	25.7±0.95b	17.9±1.13e	-30.4
	**Anthers**	**CT1**	9.6±0.98f	9.1±0.75f	- 5.2	14.3±0.85d	11.3±0.93e	-21
		**CT2**	11.3±1.15e	10.6±0.84e	- 6.2	16.5±0.95c	11.8±0.95e	-28.5
		**CS1**	11.6±0.89e	12.5±1.12de	- 7.8	20.5±0.90a	14.5±0.90d	-29.3
		**CS2**	10.5±0.90e	11.3±0.82f	- 7.6	18.9±1.19b	13.6±1.03d	-28.0
	**Ovules**	**CT1**	8.1±0.67e	8.8±0.45e	- 8.6	12.3±0.85b	9.8±0.80c	-20.3
		**CT2**	9.4±0.63d	10.4±0.87c	- 10.6	13.4±1.15b	10.3±0.96c	-23.1
		**CS1**	10.2±0.93c	9.1±0.69d	- 10.8	16.5±1.03a	12.3±1.09b	-25.5
		**CS2**	9.8±0.62d	9.6±0.76d	- 2.0	15.9±0.77a	12.9±0.69b	-18.9
*CV*	**Leaves**	**CT1**	0.23±0.014c	0.26±0.012b	13	0.19±0.011d	0.21±0.014cd	10.5
		**CT2**	0.21±0.014c	0.25±0.008b	19	0.18±0.014d	0.2±0.008d	11.1
		**CS1**	0.23±0.014c	0.27±0.011a	17.4	0.12±0.011f	0.16±0.011e	33.3
		**CS2**	0.21±0.01cd	0.25±0.014b	19	0.11±0.008f	0.17±0.011e	54.5
	**Anthers**	**CT1**	0.19±0.01d	0.23±0.008b	21.1	0.14±0.008f	0.17±0.008e	21.4
		**CT2**	0.21±0.011c	0.25±0.011a	19	0.17±0.011e	0.19±0.008d	11.8
		**CS1**	0.18±0.014d	0.23±0.008ab	27.8	0.11±0.011g	0.17±0.008e	54.5
		**CS2**	0.2±0.014c	0.24±0.014a	20	0.12±0.008g	0.18±0.014d	50
	**Ovules**	**CT1**	0.21±0.011b	0.24±0.014a	14.3	0.17±0.011c	0.2±0.008b	17.6
		**CT2**	0.2±0.014b	0.25±0.014a	25	0.16±0.008c	0.19±0.014b	18.8
		**CS1**	0.19±0.011b	0.22±0.014ab	15.8	0.1±0.023d	0.17±0.008c	70
		**CS2**	0.2±0.014b	0.24±0.014a	20	0.09±0.004e	0.16±0.012c	63.3
*MDA*	**Leaves**	**CT1**	11.3±1.15f	12.8±1.71e	-13.3	18.4±1.12d	15.3±1.15e	-16.8
		**CT2**	13.2±1.50e	14.3±1.15e	-8.3	20.5±1.15c	16.4±1.04de	-20
		**CS1**	12.9±1.16ef	14.3±0.66e	-10.9	27.9±1.13b	17.9±1.21d	-35.8
		**CS2**	13.8±1.21e	14.6±0.91e	-5.8	29.4±1.64a	21.4±1.15c	-27.2
	**Anthers**	**CT1**	9.6±0.86e	10.1±1.16e	-5.2	13.6±1.21c	10.2±0.96de	-25
		**CT2**	9.1±0.80e	10.5±0.98de	-15.4	14.6±1.09c	11.5±1.12d	-21.2
		**CS1**	10.1±1.02e	12.3±1.06d	-21.8	21.5±1.15a	15.6±0.89b	-27.4
		**CS2**	8.4±0.43f	10.7±1.01de	-27.4	20.4±0.98a	14.3±0.81c	-29.9
	**Ovules**	**CT1**	9.1±0.80d	10.4±1.01c	-14.3	13.4±0.90b	10.5±1.01c	-21.6
		**CT2**	10.4±1.04c	11.4±1.03c	-9.6	14.3±0.86b	11.3±1.03c	-21
		**CS1**	10.9±0.89c	11.4±0.83c	-4.6	19.7±0.70a	14.5±1.03b	-26.4
		**CS2**	9.3±0.72d	10.9±0.98c	-17.2	18.4±1.06a	13.6±0.89b	-26.1
*H_2_O_2_*	**Leaves**	**CT1**	1.8±0.34g	1.9±0.31g	-5.6	3.2±0.32e	2.6±0.26f	-18.8
		**CT2**	2.1±0.29fg	1.9±0.28g	-9.5	3.8±0.28d	3.3±0.29e	-13.2
		**CS1**	2.3±0.31f	2.1±0.26fg	-8.7	4.8±0.26b	3.9±0.31d	-18.8
		**CS2**	2.5±0.23f	2.3±0.26f	-8	5.1±0.23a	4.3±0.29c	-15.7
	**Anthers**	**CT1**	1.3±0.26f	1.4±0.20f	-7.7	2.6±0.28c	1.9±0.23e	-26.9
		**CT2**	1.6±0.28f	1.8±0.23e	-12.5	2.5±0.20cd	1.8±0.26e	-28
		**CS1**	1.8±0.23ef	1.6±0.26ef	-11.1	3.5±0.23b	2.9±0.29c	-17.1
		**CS2**	2.1±0.23e	1.8±0.34e	-14.3	3.9±0.26a	2.8±0.31c	-28.2
	**Ovules**	**CT1**	1.1±0.26de	1.3±0.26c	-18.2	2.5±0.23b	1.9±0.31c	-24
		**CT2**	1.3±0.18d	1.6±0.20cd	-23.1	2.7±0.26b	1.8±0.31c	-33.3
		**CS1**	1.8±0.31c	1.4±0.23d	-22.2	3.1±0.23a	2.6±0.23b	-16.1
		**CS2**	1.9±0.26c	1.6±0.17cd	-15.8	3.3±0.29a	2.5±0.20b	-24.2

Values represent mean ± S.E. Different lowercase letters indicate significant differences among genotype *treatment interaction according to Tukey’s test (*P* < 0.05), within each organ (leaves, anthers, and ovules). LT, low temperature; EL, electrolyte leakage; CV, cellular viability; MDA, malonaldehyde; H_2_O_2_, hydrogen peroxide.

Cold tolerant genotypes (CT1: ICC 17258; CT2: ICC 16349); Cold sensitive genotypes (CS1: ICC 15567; CS2: GPF-2).

**Table 2:** Effect of cold stress on anti-oxidant traits across organs and contrasting Chickpea genotypes under four treatments (control, C+SNP, LT, SNP+LT) and with percentage change.

*Traits*	*Tissue*	*Genotypes*	*Control*	*Control+Snp*	*% Change*	*lt*	*Snp+lt*	*% change*	*Traits*	*Tissue*	*Genotypes*	*Control*	*Control+SNP*	*% change*	*lt*	*Snp+lt*	*% Change*
*SOD*	**Leaves**	**CT1**	1.76±0.22e	1.87±0.12e	6.3	2.67±0.09c	2.88±0.10b	7.9	***GR***	**Leaves**	**CT1**	1.38±0.10d	1.43±0.18c	3.6	2.41±0.22a	2.56±0.21a	6.2
		**CT2**	1.45±0.12f	1.98±0.21e	36.6	2.87±0.22b	3.13±0.22a	9.1			**CT2**	1.54±0.12c	1.65±0.19c	7.1	2.18±0.17ab	2.76±0.22a	26.6
		**CS1**	1.43±0.23e	2.13±0.26d	49.0	3.13±0.22a	2.87±0.13b	8.3			**CS1**	1.51±0.17c	1.61±0.17c	6.6	1.76±0.20c	2.18±0.19a	23.9
		**CS2**	1.26±0.14f	1.86±0.06e	47.6	2.95±0.10b	2.76±0.10c	6.4			**CS2**	1.38±0.22c	1.49±0.20c	8.0	1.69±0.18c	2.09±0.35ab	23.7
	**Anthers**	**CT1**	1.54±0.15c	1.65±0.12c	7.1	1.87±0.12b	2.14±0.15a	14.4		**Anthers**	**CT1**	1.43±0.20d	1.54±0.09d	7.7	2.11±0.21b	2.65±0.12a	25.6
		**CT2**	1.24±0.13d	1.67±0.12c	34.7	1.92±0.10b	2.31±0.22a	20.3			**CT2**	1.25±0.09e	1.39±0.08d	11.2	2.18±0.22b	2.87±0.16a	31.7
		**CS1**	1.13±0.07d	1.48±0.18c	31.0	1.87±0.12b	1.98±0.14b	5.9			**CS1**	1.38±0.10d	1.48±0.15d	7.2	1.88±0.07c	2.76±0.22a	46.8
		**CS2**	1.09±0.30d	1.42±0.21c	30.3	1.91±0.23b	2.15±0.29a	12.6			**CS2**	1.25±0.22d	1.41±0.19d	12.8	1.84±0.07c	2.59±0.16a	40.8
	**Ovules**	**CT1**	1.18±0.07e	1.34±0.12d	13.6	1.76±0.12c	2.1±0.16b	19.3		**Ovules**	**CT1**	1.18±0.08ef	1.27±0.12e	7.6	2.06±0.19b	2.45±0.09a	18.9
		**CT2**	1.27±0.09d	1.45±0.15d	14.2	1.83±0.09c	2.42±0.21a	32.2			**CT2**	1.27±0.08e	1.32±0.06e	3.9	2.16±0.16b	2.38±0.12a	10.2
		**CS1**	1.19±0.18d	1.36±0.09d	14.3	2.13±0.18b	2.05±0.25b	3.8			**CS1**	1.13±0.10ef	1.29±0.08e	14.2	1.76±0.12d	2.17±0.14b	23.3
		**CS2**	1.31±0.19d	1.48±0.17d	13.0	2.17±0.22b	2.11±0.20b	2.8			**CS2**	1.08±0.09f	1.29±0.07e	19.4	1.56±0.12d	1.98±0.14c	26.9
*CAT*	**Leaves**	**CT1**	1.38±0.15d	1.43±0.07d	3.6	2.05±0.24ab	2.34±0.14a	14.1	***ASC***	**Leaves**	**CT1**	26.3±1.15d	28.3±1.18cd	7.6	32.3±1.58cd	34.5±1.44c	6.8
		**CT2**	1.41±0.09d	1.54±0.04d	9.2	1.98±0.13b	2.19±0.10ab	10.6			**CT2**	29.4±1.38c	30.9±0.98c	5.1	36.7±1.07b	38.4±1.09a	4.6
		**CS1**	1.29±0.07d	1.43±0.1d	10.9	1.76±0.09c	2.17±0.15a	23.3			**CS1**	23.6±1.48e	25.3±1.42e	7.2	30.4±1.12d	34.9±1.50c	14.8
		**CS2**	1.36±0.15d	1.49±0.18d	9.6	1.86±0.12c	2.15±0.32a	15.6			**CS2**	25.6±0.86d	26.9±1.13e	5.1	29.4±1.36d	33.7±1.37c	14.6
	**Anthers**	**CT1**	1.21±0.09cd	1.34±0.12c	10.7	1.76±0.09b	1.87±0.08b	6.3		**Anthers**	**CT1**	21.5±0.87d	25.6±1.39c	19.1	28.4±1.45b	31.4±1.42a	10.6
		**CT2**	1.26±0.12c	1.46±0.17c	15.9	1.87±0.08b	2.12±0.19a	13.4			**CT2**	22.6±1.12d	24.6±1.15c	8.8	28.3±1.33b	31.3±1.39a	10.6
		**CS1**	1.15±0.02d	1.32±0.09c	14.8	1.89±0.08b	2.18±0.25a	15.3			**CS1**	17.8±1.38f	20.5±1.38e	15.2	14.6±0.90g	19.5±0.87f	33.6
		**CS2**	1.19±0.09cd	1.31±0.12c	10.1	1.78±0.09b	2.14±0.18a	20.2			**CS2**	19.3±1.26e	22.5±0.93d	16.6	13.8±1.50h	22.3±1.45d	61.6
	**Ovules**	**CT1**	1.18±0.07de	1.28±0.12d	8.5	1.54±0.18cd	1.76±0.15c	14.3		**Ovules**	**CT1**	19.6±0.89e	22.1±1.18c	12.8	25.4±1.50b	27.6±1.49a	8.7
		**CT2**	1.26±0.24b	1.35±0.24d	7.1	1.84±0.08c	2.16±0.16a	17.4			**CT2**	21.3±1.42d	23.4±1.18c	9.9	26.7±1.44b	28.3±1.34a	6.0
		**CS1**	1.15±0.16d	1.35±0.15d	17.4	1.76±0.15c	2.11±0.21a	19.9			**CS1**	17.3±1.36f	20.4±1.45d	17.9	14.5±0.89h	20.4±1.42d	40.7
		**CS2**	1.09±0.09e	1.28±0.16d	17.4	1.69±0.09cd	2.32±0.22a	37.3			**CS2**	15.7±0.92g	19.2±1.07e	22.3	13.5±1.47h	19.4±0.83e	43.7
*APX*	**Leaves**	**CT1**	1.38±0.15d	1.43±0.09d	3.6	2.41±0.14b	2.56±0.18a	6.2	***GSH***	**Leaves**	**CT1**	13.6±0.85f	15.3±0.60d	12.5	17.1±0.95bc	19.2±0.76a	12.3
		**CT2**	1.54±0.09c	1.65±0.12c	7.1	2.18±0.16b	2.76±0.18a	26.6			**CT2**	17.2±0.86bc	16.9±0.72c	1.7	17.8±1.09b	18.2±0.78b	2.2
		**CS1**	1.51±0.12c	1.61±0.12c	6.6	1.76±0.09c	2.18±0.15b	23.9			**CS1**	15.9±0.77cd	16.8±1.10c	5.7	18.2±1.35ab	20.3±1.12a	11.5
		**CS2**	1.38±0.15d	1.49±0.18d	8.0	1.69±0.09c	2.09±0.32b	23.7			**CS2**	12.7±1.27f	14.3±0.63e	12.6	15.4±0.63d	18.3±0.69b	18.8
	**Anthers**	**CT1**	1.43±0.17e	1.54±0.15e	7.7	2.11±0.19c	2.65±0.19b	25.6		**Anthers**	**CT1**	14.3±0.63d	15.3±0.90d	7.0	17.1±0.89c	19.2±0.82a	12.3
		**CT2**	1.25±0.12f	1.39±0.12f	11.2	2.18±0.25c	2.87±0.13a	31.7			**CT2**	11.4±0.84ef	13.4±1.15de	17.5	16.1±0.84c	18.7±0.87b	16.1
		**CS1**	1.38±0.09f	1.48±0.09e	7.2	1.88±0.08d	2.76±0.10b	46.8			**CS1**	10.4±0.92f	12.4±0.93e	19.2	8.2±0.63h	11.1±0.99f	35.4
		**CS2**	1.25±0.08f	1.41±0.09f	12.8	1.84±0.08d	2.59±0.14c	40.8			**CS2**	12.3±1.18e	13.9±0.89de	13.0	9.2±0.81g	11.4±0.92f	23.9
	**Ovules**	**CT1**	1.18±0.06f	1.27±0.12e	7.6	2.06±0.20b	2.45±0.19a	18.9		**Ovules**	**CT1**	11.1±1.13de	13.2±1.20d	18.9	15.4±0.63b	17.3±0.78a	12.3
		**CT2**	1.27±0.25e	1.32±0.13e	3.9	2.16±0.26a	2.38±0.22a	10.2			**CT2**	12.3±1.18d	12.9±0.86d	4.9	14.1±0.64c	16.5±0.80b	17.0
		**CS1**	1.13±0.16e	1.37±0.15e	21.2	1.76±0.15c	2.14±0.21b	21.6			**CS1**	9.9±0.75e	11.3±0.85de	14.1	8.3±0.60f	10.9±1.00e	31.3
		**CS2**	1.08±0.09g	1.29±0.16e	19.4	1.56±0.18c	1.98±0.27c	26.9			**CS2**	8.6±0.47f	10.4±0.72e	20.9	7.4±0.7g	9.4±0.52ef	27.0

Values represent mean  ± S.E. Different lower-case letters indicate a significant difference among genotype * treatment interaction according to Tukey’s test (*P* < 0.05), within each organ. LT, low temperature; SOD, Superoxide dismutase; CAT, catalase; APX, ascorbate peroxidase; GR, glutathione reductase; ASC, ascorbic acid; GSH, reduced glutathione.

Cold tolerant genotypes (CT1: ICC 17258; CT2: ICC 16349); Cold sensitive genotypes (CS1: ICC 15567; CS2: GPF-2).

**Table 3:** Effect of cold stress on cryoprotectants in various organs and contrasting chickpea genotypes under four treatments (control, control+SNP, LT, SNP+LT) and with percentage change.

Traits	Tissue	Genotypes	Control	Control+SNP	% Change	lt	Snp+lt	% change
Pro	**Leaves**	**CT1**	16.3±0.87ij	17.7±1.02i	8.6	28.3±0.60d	33.4±1.42a	18.0
		**CT2**	18.1±0.77i	19.1±0.89h	5.5	24.5±0.63f	29.1±0.80c	18.8
		**CS1**	14.9±0.55l	16.2±0.64j	8.7	24.5±0.87f	31.3±0.72b	27.8
		**CS2**	15.6±0.69k	17.3±0.89ij	10.9	21.4±0.98g	26.7±1.10e	24.8
	**Anthers**	**CT1**	11.9±1.02ef	12.8±0.86ef	7.6	16.5±0.57d	19.8±1.04a	20
		**CT2**	13.4±0.96e	14.4±0.57e	7.5	18.7±0.75b	20.1±0.90a	7.5
		**CS1**	12.6±0.89ef	13.8±0.89e	9.5	10.8±0.81f	14.5±0.90e	34.3
		**CS2**	10.6±0.95f	12.1±0.61ef	14.2	8.6±0.43g	17.8±1.04c	107
	**Ovules**	**CT1**	10.4±0.57e	11.4±0.85e	9.6	15.9±0.89c	18.7±0.74b	17.6
		**CT2**	9.5±0.77ef	10.8±0.95e	13.7	14.3±0.89d	20.6±0.73a	44.1
		**CS1**	8.7±0.35g	9.7±0.75e	11.5	6.9±0.57h	14.5±3.31e	110.1
		**CS2**	9.1±0.71ef	10.8±0.92e	18.7	5.8±0.56i	15.6±0.99c	169.0
Treh	**Leaves**	**CT1**	5.9±0.37d	6.2±0.32c	5.1	7.4±0.36b	7.9±0.40b	6.8
		**CT2**	6.2±0.29c	6.6±0.31c	6.5	8.1±0.23b	8.9±0.40a	9.9
		**CS1**	5.8±0.27e	6.3±0.37c	8.6	4.3±0.31f	5.6±0.35d	30.2
		**CS2**	5.3±0.29e	5.9±0.26d	11.3	4.1±0.35f	5.3±0.31e	29.3
	**Anthers**	**CT1**	4.7±0.49d	5.1±0.31d	8.5	6.2±0.32b	6.9±0.37a	11.3
		**CT2**	4.9±0.37d	5.6±0.31c	14.3	6.5±0.23b	7.1±0.40a	9.2
		**CS1**	3.3±0.31g	3.7±0.26f	12.1	2.1±0.29i	3.2±0.34g	52.4
		**CS2**	3.9±0.29f	4.2±0.34e	7.7	2.6±0.28h	3.7±0.31f	42.3
	**Ovules**	**CT1**	3.1±0.34de	3.6±0.23d	16.1	4.6±0.26b	5.1±0.26a	10.9
		**CT2**	2.9±0.31e	3.4±0.23d	17.2	4.1±0.32c	4.6±0.28b	12.2
		**CS1**	2.1±0.29f	2.3±0.29f	9.5	1.6±0.23g	2.1±0.40f	31.3
		**CS2**	2.5±0.23ef	2.7±0.28e	8.00	1.9±0.34fg	2.3±0.31ef	21.1
Suc	**Leaves**	**CT1**	17.4±0.66e	19.3±0.60d	10.9	22.4±1.06b	24.3±0.92a	8.5
		**CT2**	19.3±0.81d	21.2±1.01c	9.8	23.6±0.85b	25.6±1.15a	8.5
		**CS1**	14±0.89g	16.4±0.95f	17.1	12.4±0.90h	16.7±0.97f	34.7
		**CS2**	12.9±0.86h	13.8±0.75g	7.0	10.5±0.81i	16.1±0.84f	53.3
	**Anthers**	**CT1**	15.8±0.86e	17.2±0.49d	8.9	19.2±1.01c	22.3±1.15ab	16.1
		**CT2**	17.2±0.83d	18.3±0.86c	6.4	21.2±1.07b	23.4±0.90a	10.4
		**CS1**	12.9±0.57g	14.3±0.63f	10.9	10.8±0.72h	13.4±0.93g	24.1
		**CS2**	11.1±0.64h	12.3±0.88g	10.8	9.6±0.49i	11.4±0.60h	18.8
	**Ovules**	**CT1**	12.6±1.3c	13.4±0.95c	6.3	14.6±0.66b	16.7±1.53a	14.4
		**CT2**	10.6±0.80d	11.9±0.86d	12.3	12.9±0.86c	14.5±0.84b	12.4
		**CS1**	8.1±0.60e	8.8±0.75e	8.6	6.7±0.33f	8.9±0.43e	32.8
		**CS2**	8.9±0.72e	9.1±0.54e	2.2	7.3±0.44f	9.1±0.56e	24.7

Values represent mean ± S.E. Different lowercase letters indicate significant differences among genotype * treatment interaction according to Tukey’s test (*P* < 0.05), within each organ. LT, low temperature; Pro, proline; Treh, trehalose; Suc, sucrose.

Cold tolerant genotypes (CT1: ICC 17258; CT2: ICC 16349); Cold sensitive genotypes (CS1: ICC 15567; CS2: GPF-2).

**Table 4:** Effect of cold stress on reproductive and yield traits in contrasting chickpea genotypes under the treatment condition (control, Control+SNP, LT, SNP+LT) and with percentage change.

*Traits*	*Genotypes*	*Control*	*Control+SNP*	*%change*	*LT*	*SNP+LT*	*% change*
*PV*	**CT1**	82.6± 2.32b	85.6± 2.06b	3.6	64.7± 2.31g	73.5± 2.63e	13.6
	**CT2**	84.7± 2.31b	88.2± 1.81a	4.1	68.2± 2.6f	76.8± 1.73d	12.6
	**CS1**	72.4± 2.64e	78.2± 2.29c	8.0	54.5± 2.32h	69.4± 1.44f	27.3
	**CS2**	76.7± 1.73d	81.3± 2.06b	6.0	51.5± 2.66i	66.3± 2.30f	28.7
*PG*	**CT1**	80.4± 2.57b	83.2± 2.58a	3.5	64.2± 2.94g	75.2± 2.22d	17.1
	**CT2**	84.6± 2.35a	82.4± 2.67a	2.6	60.4± 2.91i	71.4± 2.02e	18.2
	**CS1**	78.3± 3.2c	83.4± 2.39a	6.5	43.5± 2.90j	63.5± 2.66h	46
	**CS2**	81.4± 2.57b	84.5± 2.32a	3.8	40.6± 2.57j	69± 1.96f	70
*SR*	**CT1**	4.3± 0.28a	4.2± 0.32a	2.3	3.5± 0.29c	4.1± 0.23a	17.1
	**CT2**	4.1± 0.32a	4.3± 0.31a	4.9	3.2± 0.32c	3.9± 0.31b	21.9
	**CS1**	3.9± 0.37b	4.1± 0.26a	5.1	2.1± 0.34e	3.1± 0.34c	47.6
	**CS2**	4.1± 0.26a	4.2± 0.32a	2.4	2.4± 0.26d	3.2± 0.31c	33.3
*OV*	**CT1**	4.1± 0.23a	4.3± 0.29a	4.9	3.4± 0.28b	3.9± 0.17a	14.7
	**CT2**	4.3± 0.29a	4.3± 0.26a	0	3.6± 0.24b	4.1± 0.32a	13.9
	**CS1**	4.2± 0.32a	4.3± 0.31a	2.4	2.6± 0.20c	3.7± 0.26b	42.3
	**CS2**	4.3± 0.29a	4.2± 0.37a	2.3	2.9± 0.60c	3.8± 0.49b	31.0
*PN*	**CT1**	18.4±2.03c	20.3±1.48b	10.3	11.2±1.18g	15.4±1.06e	37.5
	**CT2**	19.3±1.01c	22.4±1.27a	16.1	9.8±0.89h	14.6±0.87f	49
	**CS1**	17.5±1.42c	20.3±1.18c	16.0	2.5±0.23k	5.6±0.34j	124
	**CS2**	15.9±0.75e	16.7±0.83d	5.0	2.1±0.32k	6.1±0.37i	190.5
*SW*	**CT1**	5.9±0.35d	6.3±0.29b	6.8	3.5±0.23j	4.6±0.23g	31.4
	**CT2**	6.1±0.28c	6.9±0.26a	13.1	4.1±0.35h	5.2±0.37e	26.8
	**CS1**	4.9±0.40f	5.8±0.44d	18.4	2.1±0.32l	3.6±0.28i	71.4
	**CS2**	4.1±0.32h	5.1±0.40e	24.4	1.9±0.32m	2.9±0.31k	52.6

Values represent mean ± S.E. Different lowercase letters indicate significant differences among genotype * treatment interaction according to Tukey’s test (*P* < 0.05). LT, low temperature; PV, pollen viability; PG, pollen germination; SR, stigma receptivity; OV, ovule viability; PN, Pod number plant ^-1^; SW, Seed weight plant ^-1^.

Cold tolerant genotypes (CT1: ICC 17258; CT2: ICC 16349); Cold sensitive genotypes (CS1: ICC 15567; CS2: GPF-2).

Principal component analysis (PCA) was performed using the *ggplot2*, *factoMineR*, and *factoextra* packages to explore the relationships among traits under low temperature (LT) and SNP + LT treatments across all three tissues. The results were further validated using heatmaps generated with the *pheatmap* package (Lê et al., 2008; Wickham, 2016; Kolde, 2019; Kassambara and Mundt, 2020).

## 3 Results

### 3.1 Preliminary experiments in cold-stressed plants of cold-tolerant and cold-sensitive genotypes

This experiment evaluated endogenous NO levels and NOS and NR activities in the leaves, anthers, and ovules of CT and CS genotypes after 21 d of cold stress (as described in the Materials and Methods).

#### 3.1.1 Endogenous nitric oxide levels

Under cold stress, the CT genotype exhibited significantly higher NO accumulation across all tissues than the CS genotype (**Supplementary Figure 1A**).

#### 3.1.2 Nitric oxide synthase activity

Cold stress significantly increased NOS activity in the leaves and anthers of the CT genotype, whereas no significant change was observed in the ovules (**Supplementary Figure 1B**). Conversely, the CS genotype displayed a marked decline in NOS activity in all organs under cold stress, which correlated with the observed decline in endogenous NO levels.

#### 3.1.3 Nitrate reductase activity

Under cold stress, the CT genotype showed a significant increase in NR activity in the leaves, anthers, and ovules (**Supplementary Figure 1C**). In contrast, NR activity in the CS genotype declined, particularly in anthers.

#### 3.1.4 Testing the effect of SNP on cold-stressed cold-tolerant and cold-sensitive genotypes

A follow-up preliminary experiment assessed the effect of different SNP concentrations (0.5, 1.0, and 1.5 mM) under three low-temperature regimes (13/7°C, 15/8°C, and 18/8°C) on the pod set percentage in cold-stressed CT and CS genotypes (**Supplementary Figure S2**). Both CT and CS genotypes generally showed improved pod set when treated with SNP under cold conditions. However, at the most severe temperature regime (13/7°C), the CS genotypes exhibited limited responsiveness, consistent with the results shown in the figure.

### 3.2 In-depth studies

To further explore the role of NO in cold stress responses, both CT and CS chickpea genotypes were treated with 1 mM SNP under control and cold stress conditions. The following traits were evaluated.

#### 3.2.1 Endogenous nitric oxide levels

Cold stress significantly reduced endogenous NO levels in all tissues examined (**Figure 1**), with the most pronounced decline observed in the anthers of both CT and CS genotypes. Under non-stress conditions, SNP application markedly increased NO levels in the leaves, anthers, and ovules of both CT and CS plants, with a stronger response observed in the CS genotypes. Under cold stress, SNP similarly elevated NO levels in all organs, with a substantially greater increase in CS genotypes, particularly in the anthers.

#### 3.2.2 Relative leaf water content

Cold stress significantly decreased the RLWC in both CT and CS genotypes, with a more severe reduction in the CS genotypes (**Table 5**). Under control conditions, SNP slightly increased RLWC in CT genotypes, but no such improvement was observed in CS genotypes. However, under cold stress, SNP application enhanced the RLWC in both CT and CS genotypes, with a notably greater effect in the CS genotypes.

**Table 5:** Effect of cold stress on leaf injury traits in contrasting chickpea genotypes under 4 treatment conditions (control, control+SNP, LT, SNP+LT) and with percentage change.

*Traits*	*Genotypes*	*Control*	*Control+SNP*	*%change*	*LT*	*SNP+LT*	*% change*
*RLWC*	**CT1**	78.9± 1.38d	79.1± 1.10d	0.3	71.6± 1.03i	74.3± 1.15g	3.8
	**CT2**	81.1±1.38a	80.2± 1.09b	1.1	74.5± 0.87f	76.4± 1.08e	2.6
	**CS1**	79.2±0.78c	78.1± 1.02d	1.4	69.4± 1.01j	73.4± 0.97h	5.8
	**CS2**	80.1±1.44a	78.2± 1.07b	2.4	68.2± 1.24j	71.4± 1.04i	4.7
*SC*	**CT1**	413.5± 9.87e	498.3± 8.2a	20.5	387.6± 11.3g	401.3± 7.3f	3.5
	**CT2**	450.4± 11.8d	487.4± 4.9b	8.2	413.4± 6.68e	426.7± 8.18e	3.2
	**CS1**	401.4± 7.92f	489.3± 29.6c	21.9	254.5± 12.8i	353.4± 19.4h	38.9
	**CS2**	389.5± 16.4g	429.4± 12.9d	10.2	243.5± 9.92i	353.4± 18.3h	45.1
*Chl*	**CT1**	23.4± 1.39b	25.4±1.39a	8.5	20.1±1.04d	22.4±0.93c	11.4
	**CT2**	21.6± 1.19d	23.7± 1.27b	9.7	18.4± 1.04f	20.4± 0.81d	10.9
	**CS1**	22.7± 0.83c	25.6± 1.28a	12.8	16.6± 0.96h	19.8± 1.04e	19.3
	**CS2**	21.3± 1.04d	24.3± 0.92b	14.1	14.9± 0.89i	17.6± 0.92g	18.1
*CF*	**CT1**	0.73± 0.003b	0.76± 0.008b	4.1	0.61± 0.008e	0.69± 0.017c	13.1
	**CT2**	0.76± 0.008a	0.75± 0.014a	1.3	0.64± 0.014d	0.71± 0.011b	10.9
	**CS1**	0.72± 0.014b	0.75± 0.008b	4.2	0.48± 0.014f	0.61± 0.011e	27.1
	**CS2**	0.75± 0.008b	0.74± 0.008b	1.3	0.51± 0.008f	0.61± 0.011e	19.6

Values represent mean ± S.E. Different lowercase letters indicate significant differences among genotype * treatment interaction according to Tukey’s test (*P* < 0.05). LT, low temperature; RLWC, relative leaf water content; SC, stomatal conductance; Chl, chlorophyll content; CF, chlorophyll fluorescence.

Cold tolerant genotypes (CT1: ICC 17258; CT2: ICC 16349); Cold sensitive genotypes (CS1: ICC 15567; CS2: GPF-2).

#### 3.2.3 Stomatal conductance

Cold stress significantly decreased stomatal conductance, with a greater decline in the CS genotypes (**Table 5**). Under control conditions, SNP enhanced stomatal conductance in both the CT and CS genotypes. Under cold stress, SNP treatment resulted in a slight increase in stomatal conductance in CT genotypes and a more substantial improvement in CS genotypes.

#### 3.2.4 Chlorophyll content

Leaf chlorophyll content declined under cold stress, with a more pronounced reduction in the CS genotypes (**Table 5**). Under control conditions, SNP application increased the chlorophyll content in both the CT and CS genotypes. When applied under cold stress, SNP further enhanced the chlorophyll content, particularly in the CS genotypes.

#### 3.2.5 Chlorophyll fluorescence

Cold stress reduced chlorophyll fluorescence in the CT and CS genotypes, with a greater decline observed in the CT genotypes (**Table 5**). Under cold stress, SNP improved chlorophyll fluorescence in both CT and CS genotypes, particularly in the CS genotypes.

#### 3.2.6 Electrolyte leakage

Cold stress significantly increased EL in the leaves, anthers, and ovules of both CT and CS genotypes (**Table 1**). Among the tissues, the leaves exhibited the highest increase in EL, particularly in the CS genotype. SNP treatment under cold stress significantly reduced EL in all tissues, with ovules showing the greatest reduction, particularly in the CS genotypes.

#### 3.2.7 Cellular viability

Cold stress significantly decreased cellular viability in the leaves, anthers, and ovules of both CT and CS genotypes, with a more pronounced decline in the CS genotypes (**Table 1**). Ovules were the most adversely affected, particularly in the CS genotypes. SNP application under cold stress improved cellular viability across all tissues, with CS genotypes showing a more robust recovery. While leaves and anthers showed moderate improvements, ovules exhibited the most significant recovery in cellular viability, particularly in the CS genotypes.

#### 3.2.8 Oxidative stress and antioxidant responses

##### 3.2.8.1 Malondialdehyde

Cold stress significantly increased MDA levels in the leaves, anthers, and ovules of both CT and CS genotypes (**Table 1**). The increase was more pronounced in the CS genotypes, with leaves exhibiting the highest levels, followed by anthers and ovules. SNP treatment under cold stress significantly reduced MDA accumulation in all organs, with the greatest reduction observed in the CS genotypes. Among the organs, leaves, and anthers responded more effectively to SNP treatment than did the ovules.

##### 3.2.8.2 Hydrogen peroxide

Cold stress markedly increased the H_2_O_2_ levels in all the organs (**Table 1**). Both CT and CS genotypes showed elevated levels, particularly in the anthers and ovules. Under control conditions, SNP had a contrasting effect, slightly increasing H_2_O_2_ levels in CT genotypes while decreasing them in CS genotypes. However, under cold stress, SNP consistently reduced H_2_O_2_ levels in all organs, with a more substantial reduction observed in CT, particularly in anthers and ovules.

##### 3.2.8.3 Superoxide dismutase activity

Cold stress significantly upregulated SOD activity in both vegetative and reproductive organs, with CS genotypes exhibiting a sharper increase (**Figure 2**, **Table 2**). The leaves showed the highest SOD induction. Under cold stress, SNP further enhanced SOD activity, particularly in the CS genotypes. Among the organs, leaves, and ovules showed a more consistent and pronounced.

##### 3.2.8.4 Catalase activity

Catalase activity increased across all organs under cold stress, with a more substantial rise observed in the CS genotypes than in the CT genotypes (**Figure 3**, **Table 2**). Anthers and ovules showed greater increases than leaves, particularly in the CS genotypes. SNP application under cold stress further enhanced CAT activity in all organs, with the most pronounced effects observed in the CS genotypes. The strongest enhancement was observed in ovules, followed by anthers and leaves.

##### 3.2.8.5 Ascorbate peroxidase activity

Cold stress increased APX activity in all examined organs in both CT and CS genotypes, with ovules showing the strongest response, particularly in the CT genotypes (**Figure 4**, **Table 2**). However, CS genotypes showed a relatively lower induction of APX activity under cold stress conditions. SNP treatment further boosted APX activity in all organs, with the greatest enhancement observed in the anthers. Notably, CS genotypes showed a more marked benefit from the SNP application.

##### 3.2.8.6 Glutathione reductase activity

Cold stress upregulated GR activity in all organs, with the anthers and ovules showing the greatest changes (**Figure 5**, **Table 2**), reflecting their increased oxidative stress vulnerability. While the CT genotypes maintained relatively higher baseline GR levels, the CS genotypes responded more strongly to SNP treatment. SNP application under cold stress markedly enhanced GR activity, particularly in the anthers of CS genotypes.

##### 3.2.8.7 Ascorbic acid

Cold stress induced organ-specific changes in ASC levels (**Figure 6**, **Table 2**). Leaves showed moderate increases in the CT and CS genotypes, whereas the anthers and ovules of the CS genotypes exhibited notable declines. In contrast, CT genotypes maintained higher Asc levels across all organs. SNP treatment significantly increased Asc content, particularly in the anthers and ovules of the CS genotypes.

##### 3.2.8.8 Reduced glutathione

Under cold stress, GSH levels showed genotype- and organ-specific responses (**Figure 7**, **Table 2**). In the CT genotypes, GSH levels increased in all organs. In contrast, CS genotypes exhibited a moderate increase in GSH levels in leaves, but significant reductions in anthers and ovules. SNP application under cold stress enhanced GSH accumulation in all organs, with the most pronounced increases observed in the anthers and ovules of the CS genotypes.

#### 3.2.9 Cryoprotectants

##### 3.2.9.1 Proline

Cold stress triggered an increase in proline levels in the leaves and anthers of CT genotypes (**Figure 8**, **Table 3**). In contrast, CS genotypes exhibited a notable reduction in proline content in the reproductive tissues, particularly the ovules. SNP application under cold stress conditions significantly enhanced proline accumulation across all organs, with a more pronounced effect in the CS genotypes. Among the organs assessed, ovules were the most adversely affected by cold stress in the CS genotypes, showing the steepest decline in proline levels. However, they also demonstrated the strongest recovery after SNP treatment.

##### 3.2.9.2 Trehalose

Cold stress increased trehalose content in all organs of CT genotypes but markedly reduced trehalose content in CS genotypes, especially in the anthers and ovules (**Figure 9**, **Table 3**). SNP application effectively alleviated these reductions and promoted trehalose accumulation in the CS and CT genotypes, with a more pronounced effect observed in the CS genotype. Anthers benefited the most from SNP treatment in terms of trehalose restoration.

##### 3.2.9.3 Sucrose

Under cold stress, the sucrose content showed genotype- and organ-specific responses (**Figure 10**; **Table 3**). In CS genotypes, ovules exhibited the most substantial decline, followed by leaves and anthers. In contrast, the CT genotypes showed moderate increases in sucrose content across all organs under cold stress. SNP application significantly mitigated sucrose depletion in CS genotypes and further enhanced sucrose accumulation in CT genotypes. Among the organs, leaves exhibited the greatest recovery and overall increase in sucrose content after SNP treatment, with anthers and ovules also showing considerable improvements, particularly in the CS genotype.

#### 3.2.10 Reproductive function

Cold stress negatively impacted key reproductive traits in both CT and CS genotypes, with more pronounced effects observed in the CS genotypes (**Table 4**). Cold stress significantly decreased pollen viability, pollen germination, stigma receptivity, and ovule viability, with CT genotypes exhibiting moderate declines and CS genotypes exhibiting severe impairments.

SNP application significantly alleviated cold-induced reproductive injuries. Both CT and CS genotypes exhibited improved pollen viability and germination following SNP treatment, with greater recovery in the CS genotypes. Stigma receptivity and ovule viability were also enhanced by SNP, with more pronounced benefits in the CS genotypes.

#### 3.2.11 Yield traits

**Figure 11:**
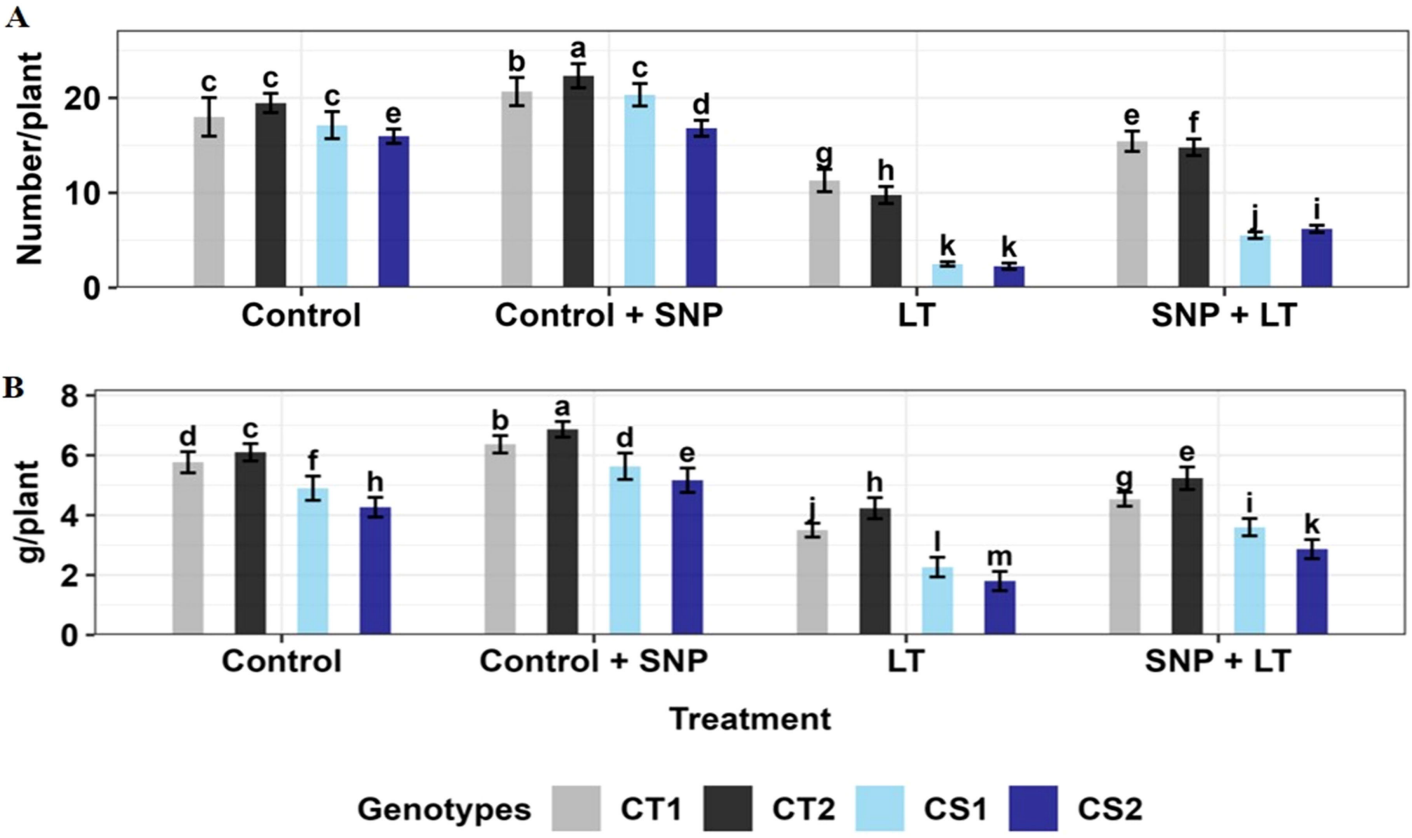
**(A)** Pod number and **(B)** Seed weight of four contrasting genotypes (CT1: ICC 17258, CT2: ICC 16349, CS1: ICC 15567, CS2: GPF-2) under different treatments: Control, Control + SNP, Low Temperature (LT), and SNP + LT. Data represent mean + SE (n =3). Different lowercase letters indicate significant differences among genotype *treatment interaction according to Tukey's test (p<0.05).

Cold stress caused a significant decline in reproductive output in both CT and CS genotypes, with the CS genotypes showing greater sensitivity (**Figure 11**; **Table 4**). The number of pods per plant decreased markedly under cold stress, with the sharpest reduction observed in the CS genotypes. Similarly, the seed weight per plant decreased under cold stress, with a more severe decline in the CS genotype than in the CT genotype. Under control conditions, SNP application modestly improved pod number and seed weight in the CT and CS genotypes. However, under cold stress, SNP markedly mitigated the yield losses. The CS genotypes showed greater relative improvement, particularly in seed weight, which exhibited the most significant recovery compared with untreated cold-stressed plants.

### 3.3 Principal component analysis and heat map

#### 3.3.1 Leaves

The PCA of the 24 leaf traits under LT and SNP + LT explained 87.8% of the variation (**Supplementary Figure 3A**). PC1 (81.3%) separated stress-protective traits (endogenous NO and chlorophyll fluorescence) from damage indicators (MDA and H_2_O_2_). PC2 (6.5%) was mainly influenced by trehalose and SOD. Heat maps and clustering (**Supplementary Figure 3B**) showed that the CS genotypes under LT were grouped with high EL, MDA, and H_2_O_2_, which decreased after SNP treatment. CT genotypes clustered with higher protective traits, particularly under SNP + LT.

#### 3.3.2 Anthers

The PCA of the 20 anther traits explained 94.2% of the variation (**Supplementary Figure 4A**). PC1 (80.5%) distinguished reproductive success traits (pollen viability and stigma receptivity) from stress markers (MDA and H_2_O_2_). PC2 (13.7%) reflected the antioxidant enzyme activities (CAT, APX, and GR). Heat maps (**Supplementary Figure 4B**) confirmed that the CS genotypes under LT clustered with oxidative stress traits, whereas SNP shifted them toward improved reproductive function. The CT genotype showed stronger antioxidant clustering under SNP + LT conditions.

#### 3.3.3 Ovules

The PCA of the 20 ovule traits explained 93% of the variation (**Supplementary Figure 5A**). PC1 (82.1%) separated protective metabolites (proline and NO) from stress damage (MDA and EL). PC2 (10.9%) was associated with antioxidant enzymes (CAT and SOD). Heat maps (**Supplementary Figure 5B**) indicated that CS genotypes under LT clustered with high oxidative stress, which SNP reduced, while CT genotypes under SNP + LT clustered with stronger antioxidant responses.

The corresponding loading values for each trait and organ are listed in **Supplementary Table 4**. Across leaves, anthers, and ovules, PCA consistently showed that SNP reduced oxidative damage in CS genotypes and enhanced protective responses in CT genotypes, underscoring SNP’s role of SNP in mitigating cold-induced reproductive injury.

## 4 Discussion

Cold stress during reproductive development is a critical constraint in chickpeas, leading to impaired reproductive function and yield loss (Croser et al., 2003; Clarke and Siddique, 2004; Kaur et al., 2022). In this study, we demonstrated that nitric oxide (NO), supplied through sodium nitroprusside (SNP), significantly improved cold tolerance during the reproductive stage. Across multiple organs—leaves, anthers, and ovules—SNP enhanced antioxidant activity, reduced oxidative stress markers, promoted the accumulation of cryoprotectants, and improved reproductive traits. These coordinated responses contribute to higher pollen viability, ovule function, and pod set under low temperatures, particularly in cold-sensitive (CS) genotypes. Our findings align with earlier reports on the protective roles of NO under abiotic stress (Cai et al., 2015; Sami et al., 2021; Kaya et al., 2022) but extend this knowledge by establishing its importance in safeguarding reproductive resilience under cold stress in chickpeas.

### 4.1 Elucidating the mechanisms of NO underlying cold stress mitigation

#### 4.1.1 Nitric oxide-mediated alleviation of cold stress injury

Cold stress reduced endogenous NO levels in leaves, anthers, and ovules, whereas SNP supplementation restored NO levels, particularly in CS genotypes. Similar enhancements of endogenous NO levels following exogenous SNP treatment have been reported in salt-stressed wheat (Kaya et al., 2022) and mustard (Sami et al., 2021). SNP-treated plants exhibited less injury under cold stress.

Electrolyte leakage (EL), a marker of membrane damage (Bajji et al., 2002), increased significantly in cold-stressed plants but was markedly reduced by SNP, consistent with observations in SNP-treated tomatoes under heat stress (Siddiqui et al., 2017) and water-stressed *Cakile maritima* (Jday et al., 2016). Cellular viability, reflecting mitochondrial activity (Aslam et al., 2022), improved with SNP treatment, likely due to NO-mediated protective mechanisms (Hajihashemi, 2021; Zhang et al., 2023). Similarly, SNP reduced cold-induced damage to chlorophyll content and fluorescence, which are key indicators of photosynthetic efficiency (Cai et al., 2015), consistent with the findings in mustard (Sami et al., 2021).

Cold-induced membrane damage and photosynthetic inhibition are largely due to the accumulation of ROS (Aslam et al., 2022; Song et al., 2024). Elevated EL, reduced viability, and impaired photosynthesis observed in this study confirm oxidative injury, which was effectively alleviated by SNP treatment. Cold stress also reduced relative leaf water content (RLWC), similar to earlier reports in rice (Dong et al., 2019), due to reduced stomatal conductance (Sun et al., 2022) and impaired hydraulic capacity in the roots (Vernieri et al., 2001, *Phaseolus*). In contrast, SNP maintained a better water status, consistent with its protective effects in sunflowers (Cechin et al., 2015) and *Cakile maritima* (Jday et al., 2016).

#### 4.1.2 Nitric oxide and oxidative stress regulation

Cold stress triggered excessive ROS accumulation, as reflected by increased malondialdehyde (MDA), H_2_O_2_, and electrolyte leakage across the leaves, anthers, and ovules. These oxidative damages are consistent with earlier findings in chickpeas and other crops under chilling stress (Kumar et al., 2011; Shen et al., 2014; Song et al., 2024). SNP supplementation markedly reduced these effects, highlighting NO’s role of NO in maintaining the cellular redox balance. The alleviation of oxidative stress is strongly associated with enhanced antioxidant activity. SNP treatment increased the activities of superoxide dismutase (SOD), ascorbate peroxidase (APX), and glutathione reductase (GR), and improved the pools of ascorbic acid (ASC) and reduced glutathione (GSH). Similar NO-mediated improvements in enzymatic and non-enzymatic antioxidants have been reported in mustard (Sami et al., 2021), tomato (Zhao et al., 2011), barley (Chen et al., 2010), and wheat (Kaya et al., 2022). By lowering ROS markers and enhancing antioxidant machinery, NO effectively protects membranes, sustains the photosynthetic apparatus, and stabilizes reproductive tissues under cold stress, thereby reinforcing its role as a versatile stress mitigator.

#### 4.1.3 Nitric oxide and cryoprotectant solutes

Cold stress induces a significant accumulation of proline, likely through the upregulation of biosynthetic enzymes and release from feedback inhibition (Raza et al., 2023). Proline functions as a multifunctional cryoprotectant, contributing to osmotic adjustment, reactive oxygen species (ROS) scavenging, and stress resilience (Raza et al., 2023). SNP-treated plants accumulated even higher proline levels under cold stress, indicating a positive role of NO in proline metabolism, consistent with the findings in *Camellia sinensis* under cold stress (Wang et al., 2021).

Trehalose, a non-reducing disaccharide with known osmoprotective and cryoprotective functions (Kosar et al., 2018), also increased under cold stress and was further enhanced by SNP, similar to the results in heat-stressed wheat (Iqbal et al., 2022). Sucrose levels increased significantly under cold stress, especially in CT genotypes, corroborating observations in wheat (Crespi et al., 1991) and tomato (Li et al., 2024), where sucrose accumulation is linked to the enhanced activity of sucrose-synthesizing enzymes (Crespi et al., 1991). SNP treatment further elevated sucrose levels across all organs, in agreement with prior reports on SNP-treated cucumber seedlings under cold stress (Feng et al., 2021).

Together, the increased accumulation of proline, trehalose, and sucrose under SNP treatment highlights NO as a central regulator of osmotic adjustment and cryoprotection, thereby supporting cold tolerance in chickpeas.

### 4.2 Organ-specific responses to cold stress and SNP

Among the three organs examined, anthers were the most susceptible to cold stress, showing greater sensitivity than leaves and ovules. This vulnerability directly threatens chickpea reproduction, as reflected by significant reductions in pollen viability, pollen germination, ovule viability, and stigma receptivity, findings consistent with earlier reports (Rani et al., 2021; Sharma and Nayyar, 2014). Cold-induced decline in NO levels in anthers, coupled with oxidative stress, likely disrupts reproductive processes, leading to poor fertilization and reduced pod set.

SNP treatment alleviated these effects, with the strongest improvements observed in anthers, particularly in the CS genotypes. SNP markedly increased endogenous NO levels, reduced EL, improved cellular viability, and enhanced cryoprotectant accumulation, all of which contribute to the protection of reproductive structures (Xie et al., 2022). Although leaves and ovules were less severely affected, they also benefited from SNP treatment, showing reduced oxidative damage. Overall, the results indicate that SNP-mediated NO supply confers protection across all major organs, with critical benefits for reproductive tissues.

### 4.3 Genotypic differences in response to cold stress and SNP

CS genotypes suffered more severe cold-induced damage than CT genotypes, as shown by reduced NO levels, higher EL, and lower cellular viability, particularly in anthers and ovules. Greater cold sensitivity in plants has previously been linked to weaker antioxidant defense activation, lower carbohydrate reserves (Kumar et al., 2011; Karami-Moalem et al., 2018, chickpea), and stronger inhibition of primary photochemistry (Shen et al., 2014, rice; Song et al., 2024, tobacco).

SNP treatment was particularly effective in CS genotypes, leading to higher NO accumulation and improved physiological performance in CS genotypes. In anthers, this increase was correlated with reduced oxidative damage, improved pollen viability, and better reproductive outcomes. Similar genotype-dependent benefits of SNP have been reported in cold-stressed tomatoes (Zhao et al., 2011) and Cd-stressed barley (Chen et al., 2010).

### 4.4 Implications for reproductive success and future directions

The most significant outcome of NO application was its impact on reproductive resilience under cold stress. SNP-treated plants maintained higher pollen viability, stigma receptivity, and ovule functionality, ultimately leading to improved pod set. These findings are consistent with those of earlier studies linking NO to reproductive development and fertilization processes (Prado et al., 2004; Zafra et al., 2010). While CT genotypes generally sustained higher pod set under stress, SNP markedly improved reproductive outcomes in CS genotypes, effectively narrowing the performance gap between the two. This demonstrates NO’s role of NO as a broad regulator of reproductive success, although its efficiency may vary with the genetic background. Taken together, our results support a model in which NO enhances cold tolerance through a multifaceted strategy involving ROS detoxification, boosting cryoprotectant levels, stabilizing water status, and safeguarding reproductive organ function. These coordinated processes converge to preserve reproductive capacity and yield stability at low temperatures.

Exogenous SNP application markedly improved cold stress resilience in chickpeas, with the strongest benefits observed in cold-sensitive (CS) genotypes. SNP restored endogenous NO levels in leaves, anthers, and ovules, reducing electrolyte leakage, sustaining cellular viability, and alleviating oxidative damage in the flowers. These effects were linked to enhanced antioxidant activity (SOD, APX, GR) and higher ASC and GSH levels, which supported efficient ROS detoxification.

SNP also promoted the accumulation of proline, trehalose, and sucrose, reinforcing osmotic adjustment and cryoprotection. Reproductive tissues, especially anthers, were most vulnerable to cold stress but showed the greatest recovery under SNP treatment, resulting in improved pollen viability, stigma receptivity, ovule function, and pod set. While CT genotypes maintained better tolerance, SNP narrowed the performance gap by significantly improving reproductive outcomes in the CS lines.

Overall, SNP enhanced cold tolerance through coordinated ROS detoxification, osmolyte accumulation, and reproductive organ protection, thereby supporting chickpea yield stability. Future studies should directly evaluate photosynthetic efficiency, water status, and the molecular regulation of NO-mediated antioxidant responses.

## 5 Conclusions

This study demonstrates that SNP treatment significantly enhances cold stress resilience in chickpea plants, particularly in cold-sensitive (CS) genotypes. Exogenous SNP application effectively elevated endogenous nitric oxide (NO) levels in anthers, leaves, and ovules, with the strongest effects observed in CS genotypes, which are typically more vulnerable to oxidative damage at low temperatures. Increased NO levels were associated with reduced electrolyte leakage and improved cellular viability, indicating that SNP mitigates membrane damage and oxidative stress. Additionally, SNP treatment boosted cryoprotectant accumulation in anthers, improving their structural integrity and function, which are key factors for maintaining pollen viability and germination under cold conditions. The observed improvements in chlorophyll content and stomatal conductance further suggest that SNP contributes to enhanced photosynthetic efficiency and water use under cold stress conditions. Overall, these findings highlight SNP’s potential of SNPs as a promising tool for improving cold tolerance, reproductive success, and yield stability in chickpea crops facing increasingly variable and challenging climates.

## Data Availability

The original contributions presented in the study are included in the article/**Supplementary Material**. Further inquiries can be directed to the corresponding author.
