# Supplementary material for: Nitric oxide-mediated modulation of reproductive resilience under cold stress in chickpea
Source: Front Plant Sci. 2025 Nov 7;16:1679156. doi: 10.3389/fpls.2025.1679156 (PMC12634336; doi:10.3389/fpls.2025.1679156)
Supplement: Supplementary file 1 [file DataSheet1.docx]

**Supplementary Table 1: Analysis of variance (ANOVA) table showing mean square values for nitric oxide (NO), nitric oxide synthase (NOS), and nitrate reductase (NR) under control and cold stress conditions across different chickpea organs.**

| **Traits** | **Genotypes** | **Treatment** | **Organ** | **G*T** | **G*O** | **O*T** | **G*T*O** | **Error** |
| --- | --- | --- | --- | --- | --- | --- | --- | --- |
| **df** | 1 | 1 | 2 | 1 | 2 | 2 | 2 | 24 |
| **NO** | 407.4*** | 1.6 | 1201.1*** | 134.2*** | 19.9** | 6.7 | 17.2* | 3.3 |
| **NOS** | 28.9*** | 2.00* | 12.01*** | 11.90*** | 0.152 | 0.805 | 0.206 | 0.33 |
| **NR** | 2.05** | 0.054 | 11.6*** | 0.010 | 0.155 | 0.055 | 0.29* | 0.22 |

**Abbreviation**: NO: nitric oxide, NOS: nitric oxide synthase, NR: nitrate reductase, G: genotypes, T: treatment, O: organs. Note: ***p ≤ 0.001, **p ≤ 0.01, *p ≤ 0.05, ns = not significant

**Supplementary Table 2: Analysis of variance (ANOVA) table showing the sum of squares and mean square values for pod set percentage across different treatments of temperature and SNP.**

| **Pod set** | **Genotypes (G)** | **Treatment (T)** | **SNP** | **G*T** | **G*SNP** | **T*SNP** | **G*T*SNP** | **Error** |
| --- | --- | --- | --- | --- | --- | --- | --- | --- |
| **df** | 1 | 3 | 3 | 3 | 3 | 9 | 9 | 64 |
| **Sum of squares** | 10,240 | 42236 | 1586 | 1358 | 240 | 751 | 192 | 368 |
| **Mean square** | 10,240*** | 14079*** | 529*** | 453*** | 80 *** | 83*** | 21*** | 6 |

Note: ***p ≤ 0.001, **p ≤ 0.01, *p ≤ 0.05, ns = not significant

**Supplementary Table 3: Analysis of variance (ANOVA) table showing the mean sum of squares for various physiological, biochemical, reproductive, and yield traits across four chickpea genotypes.**

| **Traits** | **Genotypes** | **Treatment** | **Organ** | **G*T** | **G*O** | **T*O** | **G*T*O** | **Error** |
| --- | --- | --- | --- | --- | --- | --- | --- | --- |
| **df** | **3** | **3** | **2** | **9** | **6** | **6** | **18** | **96** |
| **Endo NO** | 365*** | 6536*** | 991*** | 41*** | 24*** | 43*** | 6 | 4 |
| **EL** | 62.5*** | 512.9 *** | 326.7*** | 8.6** | 1.3 | 38.3*** | 1.4 | 2.6 |
| **CV** | 0.00553*** | 0.06238*** | 0.00306** | 0.00165*** | 0.00089 | 0.00030 | 0.00032 | 0.00046 |
| **MDA** | 107.8*** | 520.2 *** | 336.4*** | 31.3*** | 8.2* | 15.4*** | 1.5 | 3.4 |
| **H_2_O_2_** | 6.12*** | 23.14*** | 13.13*** | 0.482* | 0.179 | 0.753** | 0.090 | 0.216 |
| **SOD** | 0.055 | 9.538*** | 5.648 *** | 0.166* | 0.205* | 0.551*** | 0.032 | 0.092 |
| **CAT** | 0.220* | 5.448*** | 0.187* | 0.080 * | 0.117 | 0.030 | 0.068 | 0.075 |
| **APX** | 0.877*** | 9.135*** | 0.454** | 0.220** | 0.024 | 0.086 | 0.038 | 0.081 |
| **GR** | 0.525*** | 9.968*** | 0.792*** | 0.205* | 0.029 | 0.082 | 0.035 | 0.083 |
| **ASC** | 440.6*** | 291.0*** | 1175.9*** | 34.4*** | 28.2 *** | 31.7 *** | 7.6 | 4.8 |
| **GSH** | 123.86*** | 67.02*** | 302.39*** | 14.29*** | 33.16*** | 5.52* | 6.21** | 2.36 |
| **Pro** | 144.2*** | 527.6*** | 1457.9*** | 30.3*** | 14.2*** | 73.7*** | 12.2*** | 2.7 |
| **Treh** | 52.25*** | 7.21 *** | 119.88*** | 5.34 *** | 1.54*** | 0.18 | 0.16 | 0.31 |
| **Suc** | 525.5*** | 76.6*** | 616.8*** | 22.9*** | 19.6*** | 3.3 | 1.5 | 2.1 |
|  | | | | | | | | |
| **Traits** | **Genotypes** | **Treatment** | **G*T** | **Error** |  |  |  |  |
| **df** | **3** | **3** | **9** | **30** |  |  |  |  |
| **RLWC** | 26.0 *** | 211.4 *** | 4.62 *** | 0.15 |  |  |  |  |
| **SC** | 22382 *** | 44816*** | 3152*** | 360 |  |  |  |  |
| **Chl** | 22.63*** | 98.4*** | 5.06** | 1.28 |  |  |  |  |
| **CF** | 0.016*** | 0.08*** | 0.003*** | 0.0002 |  |  |  |  |
| **PV** | 328.5*** | 1234.5*** | 16.4** | 4.4 |  |  |  |  |
| **PG** | 223.0*** | 2371.3*** | 106.2*** | 0.5 |  |  |  |  |
| **SR** | 1.50*** | 4.98*** | 0.25** | 0.07 |  |  |  |  |
| **OV** | 0.45 | 3.89*** | 0.21 | 0.23 |  |  |  |  |
| **PN** | 129.1*** | 473.5*** | 15.7*** | 2.1 |  |  |  |  |
| **SW** | 10.4*** | 21.7*** | 0.10 | 0.16 |  |  |  |  |

**Abbreviation:** Endo NO: Endogenous nitric oxide, RLWC: Relative leaf water content, SC: Stomatal conductance, Chl: Chlorophyll content, CF: Chlorophyll fluorescence, EL: Electrolyte leakage, CV: Cellular viability, MDA: Malondialdehyde, H_2_O_2_: Hydrogen peroxide, SOD: Superoxide dismutase, CAT: Catalase, APX: Ascorbate peroxidase, GR: Glutathione reductase, ASC: Ascorbic acid, GSH: Reduced glutathione, Pro: Proline, Treh: Trehalose, Suc: Sucrose, PV: Pollen viability, PG: Pollen germination, SR: Stigma receptivity, OV: Ovule viability, PN: Pod number plant^–1^, SW: Seed weight plant^–1^.

Note: ***p ≤ 0.001, **p ≤ 0.01, *p ≤ 0.05, ns = not significant.

**Supplementary Table 4: Loading matrix values from principal component analysis for various physiological, biochemical, and yield parameters of different tissues for LT and SNP+LT treatments.**

| **Traits** | **Tissue** | **Treatment** | |
| --- | --- | --- | --- |
|  |  | LT | SNP+LT |
| **Endo NO** | Leaves | 0.220 | 0.229 |
|  | Anthers | 0.236 | 0.233 |
|  | Ovules | 0.234 | 0.220 |
| **RLWC** | Leaves | 0.191 | 0.210 |
| **SC** | Leaves | 0.217 | 0.229 |
| **Chl** | Leaves | 0.205 | 0.184 |
| **CF** | Leaves | 0.211 | 0.232 |
| **EL** | Leaves | -0.200 | -0.218 |
|  | Anthers | -0.218 | -0.253 |
|  | Ovules | -0.229 | -0.239 |
| **CV** | Leaves | 0.220 | 0.216 |
|  | Anthers | 0.216 | 0.102 |
|  | Ovules | 0.234 | 0.234 |
| **MDA** | Leaves | -0.219 | -0.201 |
|  | Anthers | -0.239 | -0.249 |
|  | Ovules | -0.229 | -0.235 |
| **H_2_O_2_** | Leaves | -0.214 | -0.204 |
|  | Anthers | -0.35 | -0.258 |
|  | Ovules | -0.227 | -0.238 |
| **SOD** | Leaves | 0.182 | 0.185 |
|  | Anthers | 0.035 | 0.199 |
|  | Ovules | 0.234 | 0.141 |
| **CAT** | Leaves | 0.203 | 0.147 |
|  | Anthers | 0.038 | 0.169 |
|  | Ovules | 0.050 | 0.160 |
| **APX** | Leaves | 0.215 | 0.231 |
|  | Anthers | 0.239 | 0.092 |
|  | Ovules | 0.221 | 0.232 |
| **GR** | Leaves | 0.215 | 0.231 |
|  | Anthers | 0.239 | 0.092 |
|  | Ovules | 0.221 | 0.227 |
| **ASC** | Leaves | 0.175 | 0.162 |
|  | Anthers | 0.242 | 0.258 |
|  | Ovules | 0.232 | 0.241 |
| **GSH** | Leaves | 0.072 | 0.071 |
|  | Anthers | 0.240 | 0.256 |
|  | Ovules | 0.234 | 0.240 |
| **Proline** | Leaves | 0.167 | 0.106 |
|  | Anthers | 0.235 | 0.236 |
|  | Ovules | 0.234 | 0.226 |
| **Treh** | Leaves | 0.216 | 0.232 |
|  | Anthers | 0.242 | 0.259 |
|  | Ovules | 0.232 | 0.241 |
| **Suc** | Leaves | 0.218 | 0.233 |
|  | Anthers | 0.240 | 0.250 |
|  | Ovules | 0.232 | 0.238 |
| **PV** |  | 0.237 | 0.229 |
| **PG** |  | 0.238 | 0.225 |
| **SR** |  | 0.232 | 0.253 |
| **OV** |  | 0.232 | 0.231 |
| **PN** |  | 0.239 | 0.256 |
| **SW** |  | 0.237 | 0.232 |

**Abbreviation:** Endo NO: Endogenous nitric oxide, RLWC: Relative leaf water content, SC: Stomatal conductance, Chl: Chlorophyll content, CF: Chlorophyll fluorescence, EL: Electrolyte leakage, CV: Cellular viability, MDA: Malondialdehyde, H_2_O_2_: Hydrogen peroxide, SOD: Superoxide dismutase, CAT: Catalase, APX: Ascorbate peroxidase, GR: Glutathione reductase, ASC: Ascorbic acid, GSH: Reduced glutathione, SUC: Sucrose, Pro: Proline, Treh: Trehalose, PV: Pollen viability, PG: Pollen germination, SR: Stigma receptivity, OV: Ovule viability, SW: Seed weight plant^–1^, PN: Pod number plant^–1^, LT: Low temperature.


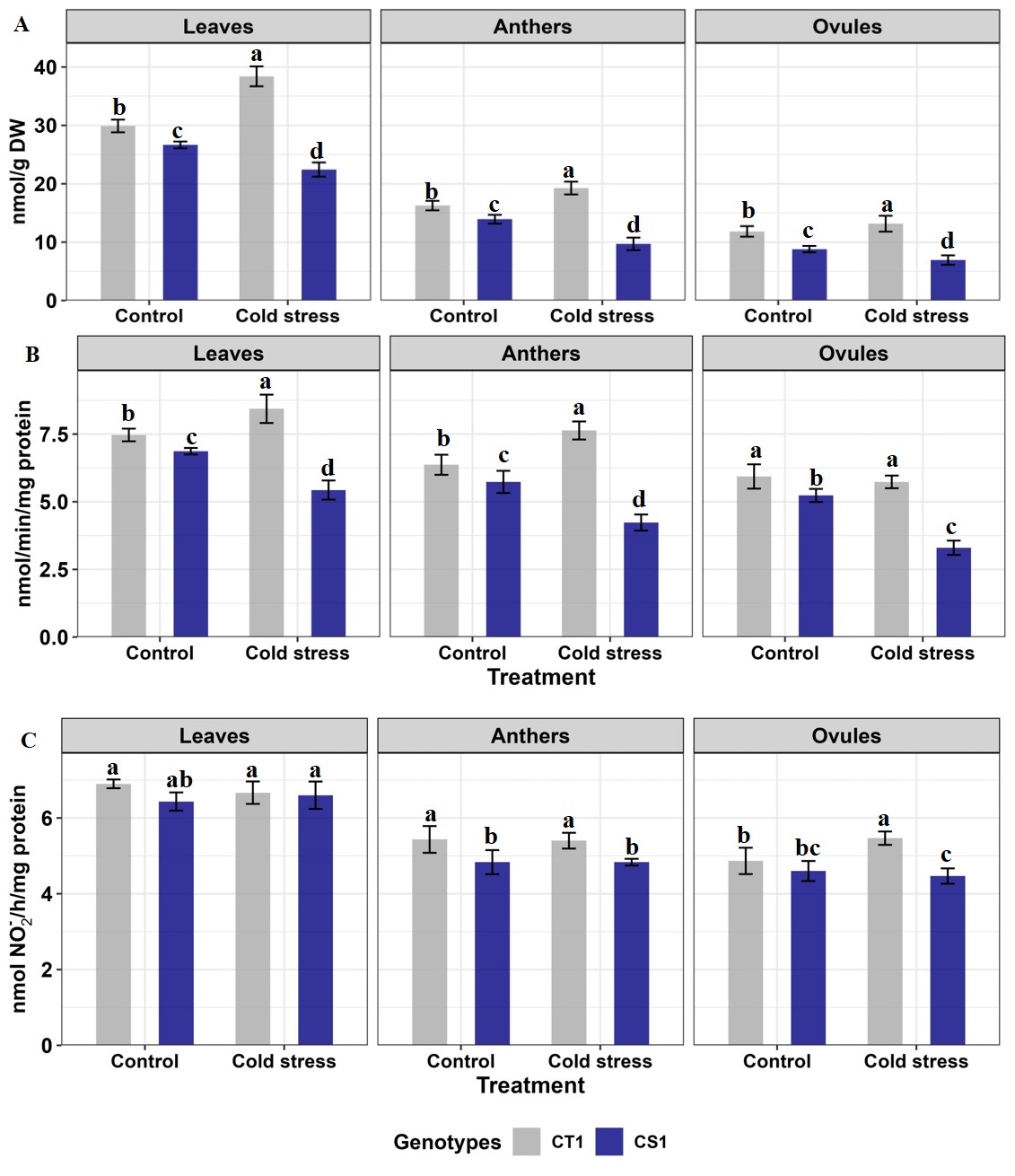


**Supplementary Figure 1.** Activities of (A) nitric oxide (NO), (B) nitric oxide synthase (NOS), and (C) nitrate reductase (NR) in leaves, anthers, and ovules of two chickpea genotypes (CT1: cold-tolerant; CS1: cold-sensitive) under control and cold stress conditions. Data represent mean ± SE (n = 3). Different lowercase letters indicate significant differences among genotype * treatment interaction according to Tukey’s test, within each organ.


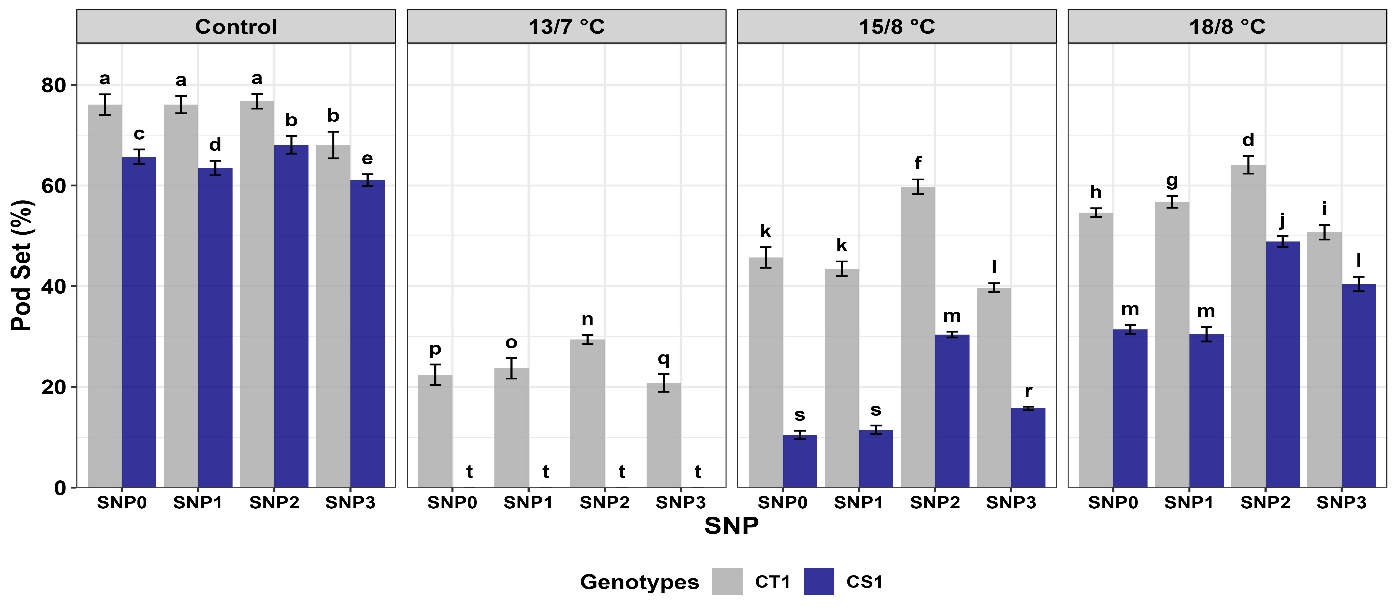


**Supplementary Figure 2.** Effect of treatments on pod set percentage in two chickpea genotypes (CT1 and CS1). Bars represent mean values ± SE (n = 3). Different lowercase letters above bars indicate statistically significant differences among genotypes, SNP levels, and treatments at P < 0.05, according to the Tukey post hoc test. Treatments include Control, 13/7 °C, 15/8 °C, and 18/8 °C, and SNP levels (SNP0 (No SNP), SNP1 (0.5mM), SNP2 (1mM), SNP3 (1.5mM).

**
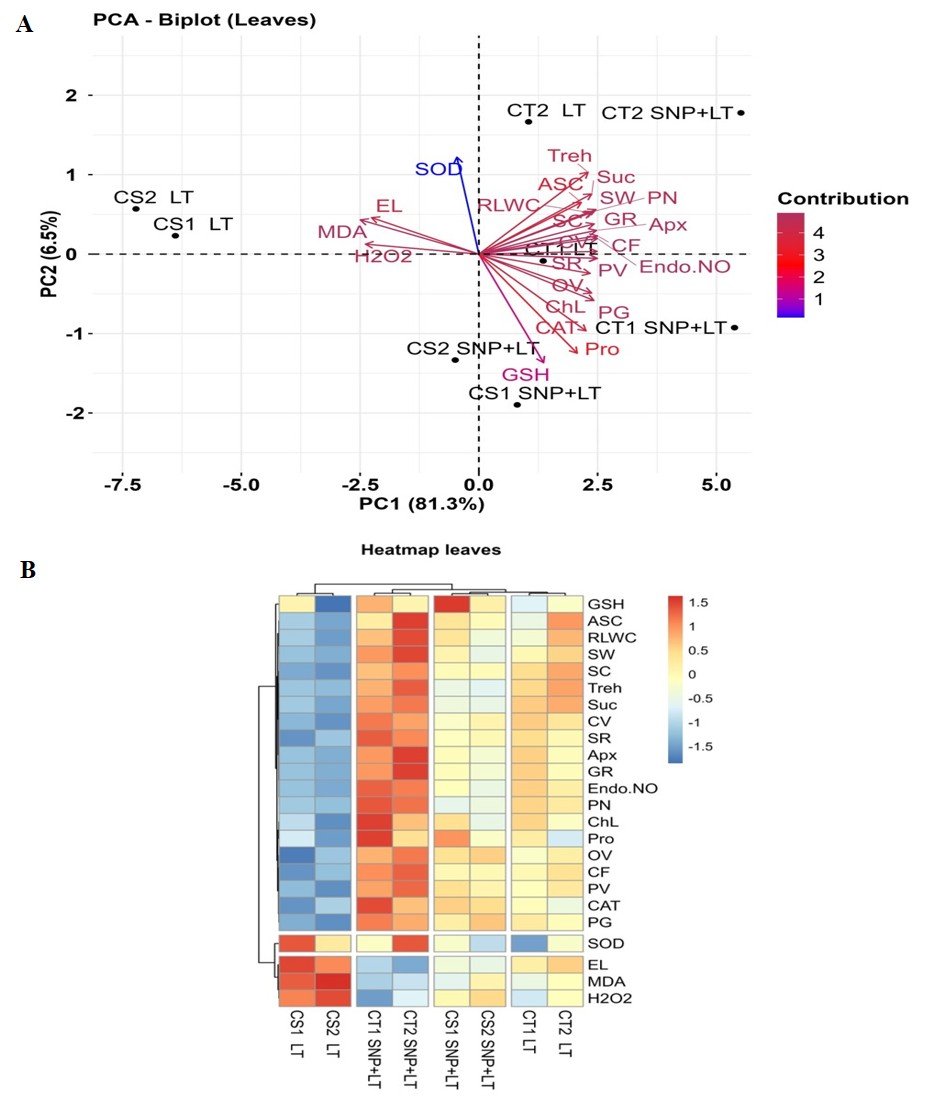
**

**Supplementary Figure 3.** Principal Component Analysis (PCA) (A); Heat Map analysis (B) of leaf traits, biochemical traits, reproductive traits, and yield traits assessed in leaf samples of chickpea genotypes under low temperature (LT) and SNP + LT conditions

**Abbreviations:** Endo No: endogenous nitric oxide, EL: electrolyte leakage, Chl: chlorophyll content, CF: chlorophyll fluorescence, RLWC: relative leaf water content, SC: stomatal conductance, CV: cellular viability, MDA: malonaldehyde, H_2_O_2_: hydrogen peroxide, SOD: superoxide dismutase, CAT: catalase, APX: ascorbate peroxidase, GR: glutathione reductase, ASC: ascorbic acid, GSH: reduced glutathione, SUC: sucrose, Pro: proline, TREH: trehalose, PV: pollen viability, PG: pollen germination, SR: stigma receptivity, OV: ovule viability SW: seed weight plant^-1^, PN: pod number plant^-1^.

**
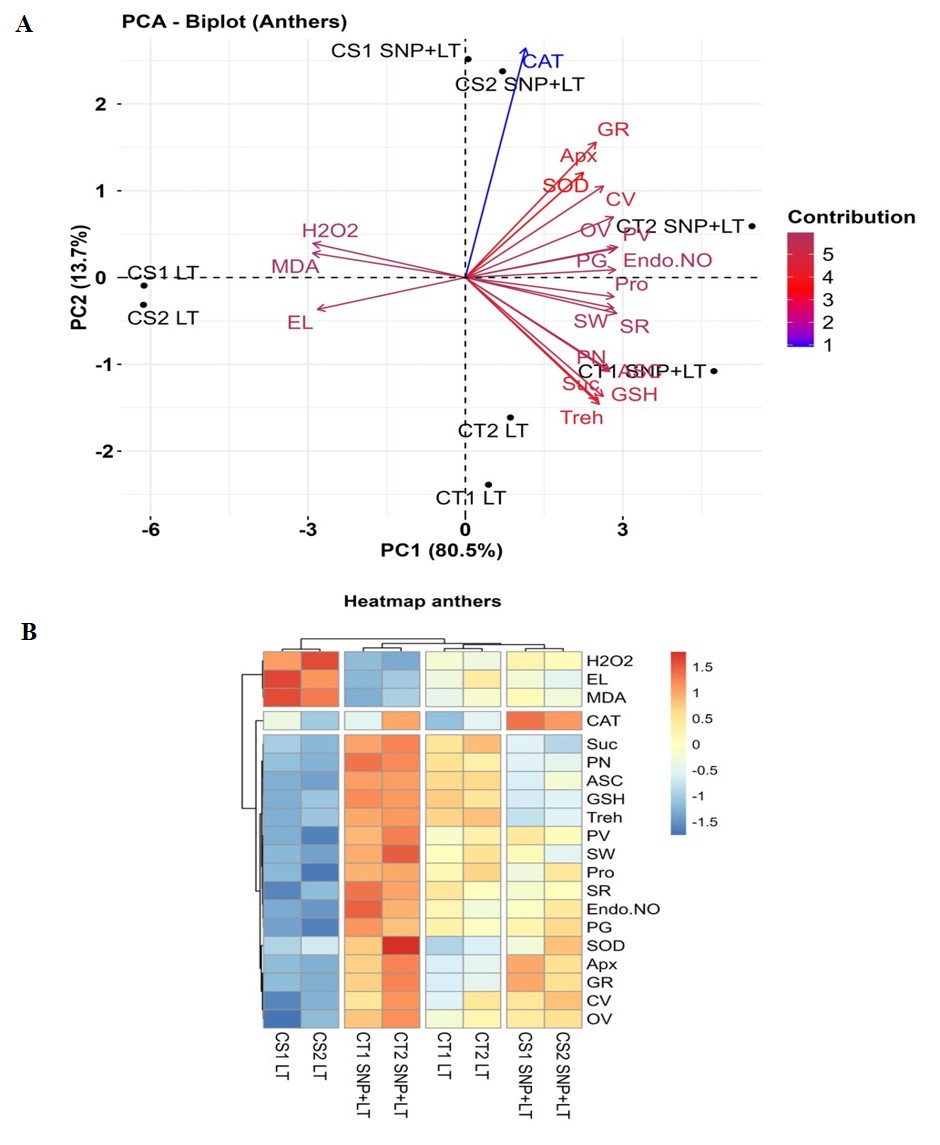
**

**Supplementary Figure 4.** Principal Component Analysis (PCA) (A); Heat Map analysis (B) of biochemical traits, reproductive traits, and yield traits assessed in anther samples of chickpea genotypes under low temperature (LT) and SNP + LT conditions

**Abbreviations:** Endo No: endogenous nitric oxide, EL: electrolyte leakage, CV: cellular viability, MDA: malonaldehyde, H_2_O_2_: hydrogen peroxide, SOD: superoxide dismutase, CAT: catalase, APX: ascorbate peroxidase, GR: glutathione reductase, ASC: ascorbic acid, GSH: reduced glutathione, SUC: sucrose, Pro: proline, TREH: trehalose, PV: pollen viability, PG: pollen germination, SR: stigma receptivity, OV: ovule viability SW: seed weight plant^-1^, PN: pod number plant^-1^.

**
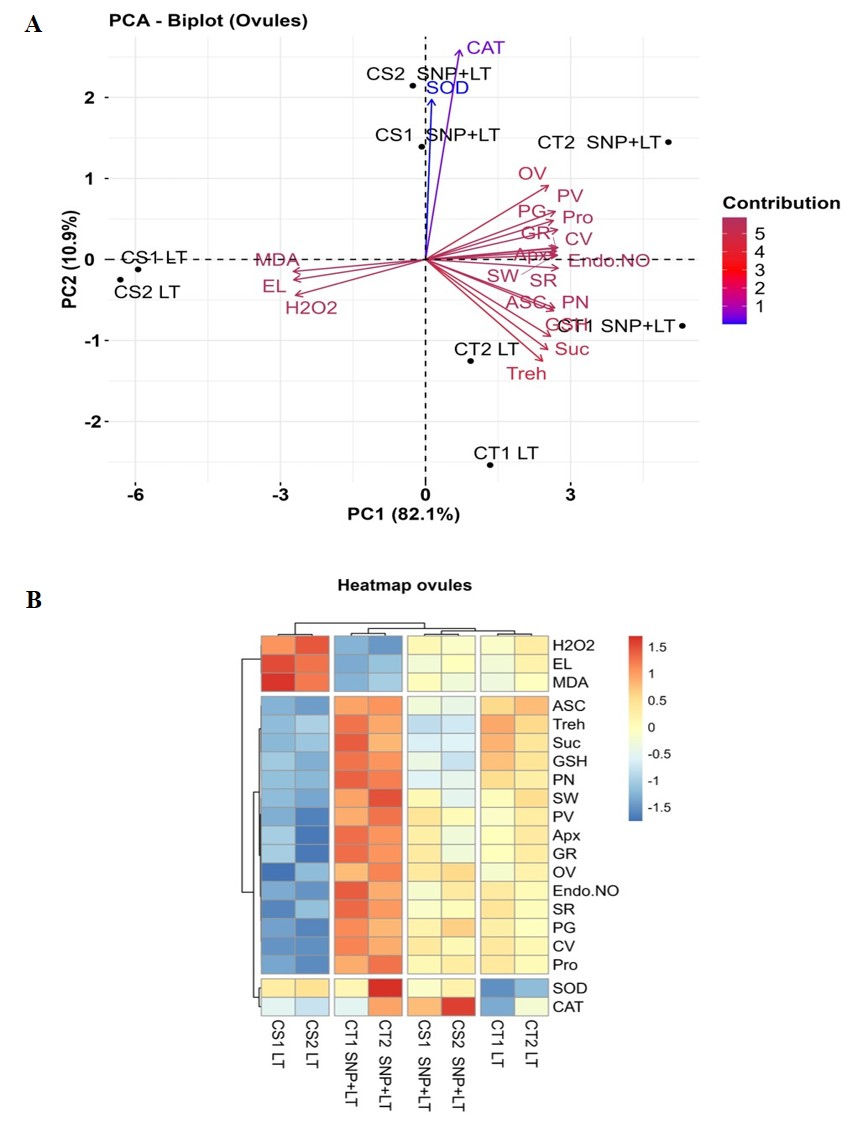
**

**Supplementary Figure 5.** Principal Component Analysis (PCA) (A); Heat Map analysis (B) of biochemical traits, reproductive traits, and yield traits assessed in ovule samples of chickpea genotypes under low temperature (LT) and SNP + LT conditions

**Abbreviations:** Endo No: endogenous nitric oxide, EL: electrolyte leakage, CV: cellular viability, MDA: malonaldehyde, H_2_O_2_: hydrogen peroxide, SOD: superoxide dismutase, CAT: catalase, APX: ascorbate peroxidase, GR: glutathione reductase, ASC: ascorbic acid, GSH: reduced glutathione, SUC: sucrose, Pro: proline, TREH: trehalose, PV: pollen viability, PG: pollen germination, SR: stigma receptivity, OV: ovule viability SW: seed weight plant^-1^, PN: pod number plant^-1^.
